# Molecular and functional characterization of chemosensory genes from the root-knot nematode *Meloidogyne graminicola*

**DOI:** 10.1186/s12864-023-09864-7

**Published:** 2023-12-06

**Authors:** Tushar K. Dutta, Voodikala S. Akhil, Manoranjan Dash, Artha Kundu, Victor Phani, Anil Sirohi

**Affiliations:** 1 Division of Nematology, ICAR-Indian Agricultural Research Institute, New Delhi, 110012 India; 2 Department of Agricultural Entomology, College of Agriculture, Uttar Banga Krishi Viswavidyalaya, Balurghat, Dakshin Dinajpur, West Bengal India

**Keywords:** Attraction, Aversion, Chemotaxis, Pluronic gel, Gene expression, DsRNA

## Abstract

**Background:**

Root-knot nematode *Meloidogyne graminicola* has emerged as a major threat in rice agroecosystems owing to climate change-induced changes in cultivation practices. Synthetic nematicides are continually being withdrawn from the nematode management toolbox because of their ill effects on the environment. A sustainable strategy would be to develop novel nematicides or resistant plants that would target nematode sensory perception, which is a key step in the host finding biology of plant-parasitic nematodes (PPNs). However, compared to the extensive literature on the free-living nematode *Caenorhabditis elegans*, negligible research has been performed on PPN chemosensory biology.

**Results:**

The present study characterizes the five chemosensory genes (*Mg-odr-7*, *Mg-tax-4*, *Mg-tax-4.1*, *Mg-osm-9*, and *Mg-ocr-2*) from *M. graminicola* that are putatively associated with nematode host-finding biology. All the genes were highly transcribed in the early life stages, and RNA interference (RNAi)-induced downregulation of each candidate gene perturbed the normal behavioural phenotypes of *M. graminicola*, as determined by examining the tracking pattern of juveniles on Pluronic gel medium, attraction to and penetration in rice root tip, and developmental progression in rice root. In addition, a detrimental effect on nematode chemotaxis towards different volatile and nonvolatile organic compounds and host root exudates was documented.

**Conclusion:**

Our findings enrich the existing literature on PPN chemosensory biology and can supplement future research aimed at identifying a comprehensive chemosensory signal transduction pathway in PPNs.

**Supplementary Information:**

The online version contains supplementary material available at 10.1186/s12864-023-09864-7.

## Background

Rice is a staple food for approximately 50% of the global population and is grown in more than 100 countries, with Asia growing 90% of the world’s rice production [1]. The productivity of rice is afflicted by a number of biotic stressors, including plant-parasitic nematodes (PPNs). Rice root-knot nematode *Meloidogyne graminicola* is becoming a major threat in rice agroecosystems, especially in Asia, where water-intensive rice agriculture (including upland and direct seeded rice) is increasingly being adopted owing to the changing environment (climate change) and socioeconomic conditions [2]. The second-stage infective juveniles (J2s) of root-knot nematodes hatch from eggs in the soil. These J2s are non-feeding and must locate a host to initiate a nematode feeding site (NFS) before their body fat reserves are depleted [3]. J2s are particularly attracted to and invade the root through the elongation zone, adjacent to the root tip. After root penetration, J2s migrate within the root to establish a hypertrophied, multinucleated NFS (popularly known as a giant cell) that serves as the recurring nutrient source for developing nematodes [4, 5]. While locating suitable hosts, J2s perceive volatile chemicals (smell) over a long distance, and local exploration involves perceiving water-soluble chemicals (taste). A blend of attractants and repellents present in the root exudates modulates nematode host-finding ability [6–8]. It was demonstrated that *M. incognita* J2s perceive gradients of volatile organic compounds to locate suitable hosts [9].

Rotation with dicots, deployment of resistant varieties, and biological control agents are traditional means of *M. graminicola* management in rice; nevertheless, each of these means has its own limitations [2, 10, 11]. Due to environmental concerns and potential health hazards, chemical nematicides are continually being withdrawn from farming practices. Several research trials are currently underway to discover and synthesize environmentally sustainable nematicides [12]. One promising strategy would be to disrupt either the nematode life cycle progression inside the host plant or its olfactory perception of the plant compounds that attract them [13, 14]. Without affecting other aspects of the environment, these methods would allow the plants to be protected from infection by root-knot nematodes [15, 16]. From a scientific perspective, these studies shed light on the need for combining molecular and pharmacological approaches for protecting plants from nematodes.

Using genetic mutation analysis and laser ablation screening, the function of a plethora of chemosensory genes has been unravelled in the model nematode *Caenorhabditis elegans*. Approximately 21 heterotrimeric G proteins, 500–1000 G protein-coupled receptors (GPCRs), and 34 guanylyl cyclase (GCYs) receptors have been deorphanized from *C. elegans* [17, 18]. Additionally, numerous chemosensory genes, including thermotaxis (*ttx*), chemotaxis (*che*, *tax2/4/6, gpa1-gpa15, daf11*), aerotaxis (*npr1*), odorant (*odr1-odr10*) defective mutants, and osmotic avoidance (*osm9*) mutants, were characterized [17, 18]. These extensive literatures led to the prediction of two major signal transduction pathways in the *C. elegans* chemoreception model: a cGMP-gated channel (cGMP production is regulated by membrane-bound GPCRs via GCY (ODR1/DAF11) followed by TAX2/TAX4 activity) and another TRPV channel (G proteins activate Gα/ODR3, downstream of which OSM9/OCR2 activate transmembrane ion transport) [17, 19].

However, the molecular basis of PPN chemoreception is yet an underexplored territory, maybe because PPNs are not amenable to forward genetic screening owing to their small size and obligate parasitic nature. RNA interference (RNAi)-based reverse genetics screening has become useful for studying gene function in PPNs [20, 21]. A handful of chemosensory genes have been characterized from PPNs such as *Mi-odr-1*, *Mi-odr-3*, *Mi-tax-2*, *Mi-tax-4* [9], *Mi-odr-7*, *Mi-odr-10*, *Mi-osm-9* [22] from *M. incognita*, and *Hg-gcy-1*, *Hg-gcy-2*, and *Hg-gcy-3* from *Heterodera glycines* [23]. The current study aims to advance that knowledge by identifying a number of chemosensory genes (such as *Mg-odr-7*, *Mg-tax-4*, *Mg-tax-4.1*, *Mg-osm-9*, and *Mg-ocr-2*) from *M. graminicola* and functionally characterizing them via extensive RNAi screens.

## Results

### Characterization of chemosensory genes from *M. graminicola*

By using 3´ and 5´ RACE-PCR and primer walking, single cDNAs harbouring the entire coding sequences of *Mg-odr-7* (NCBI Genbank accession number: OQ445600), *Mg-tax-4* (OQ445601), *Mg-tax-4.1* (OQ445603; identified as a homologue of *Mg-tax-4*), *Mg-osm-9* (OQ445605), and *Mg-ocr-2* (OQ445602) were separately obtained. The ORFs of *Mg-odr-7* (1665 bp), *Mg-tax-4* (4296 bp), *Mg-tax-4.1* (2688 bp), *Mg-osm-9* (3231 bp), and *Mg-ocr-2* (2646 bp) encode 554, 1431, 895, 1076, and 881 amino acids (aa), respectively. The predicted Mg-ODR-7, Mg-TAX-4, Mg-TAX-4.1, Mg-OSM-9, and Mg-OCR-2 proteins have a calculated molecular mass of 61.88 (isoelectric point/pI – 8.17), 165.57 (pI – 8.93), 102.98 (pI – 9.38), 123.17 (pI – 7.99), and 101.13 (pI – 8.41) kDa, respectively.

**Fig. 1:**
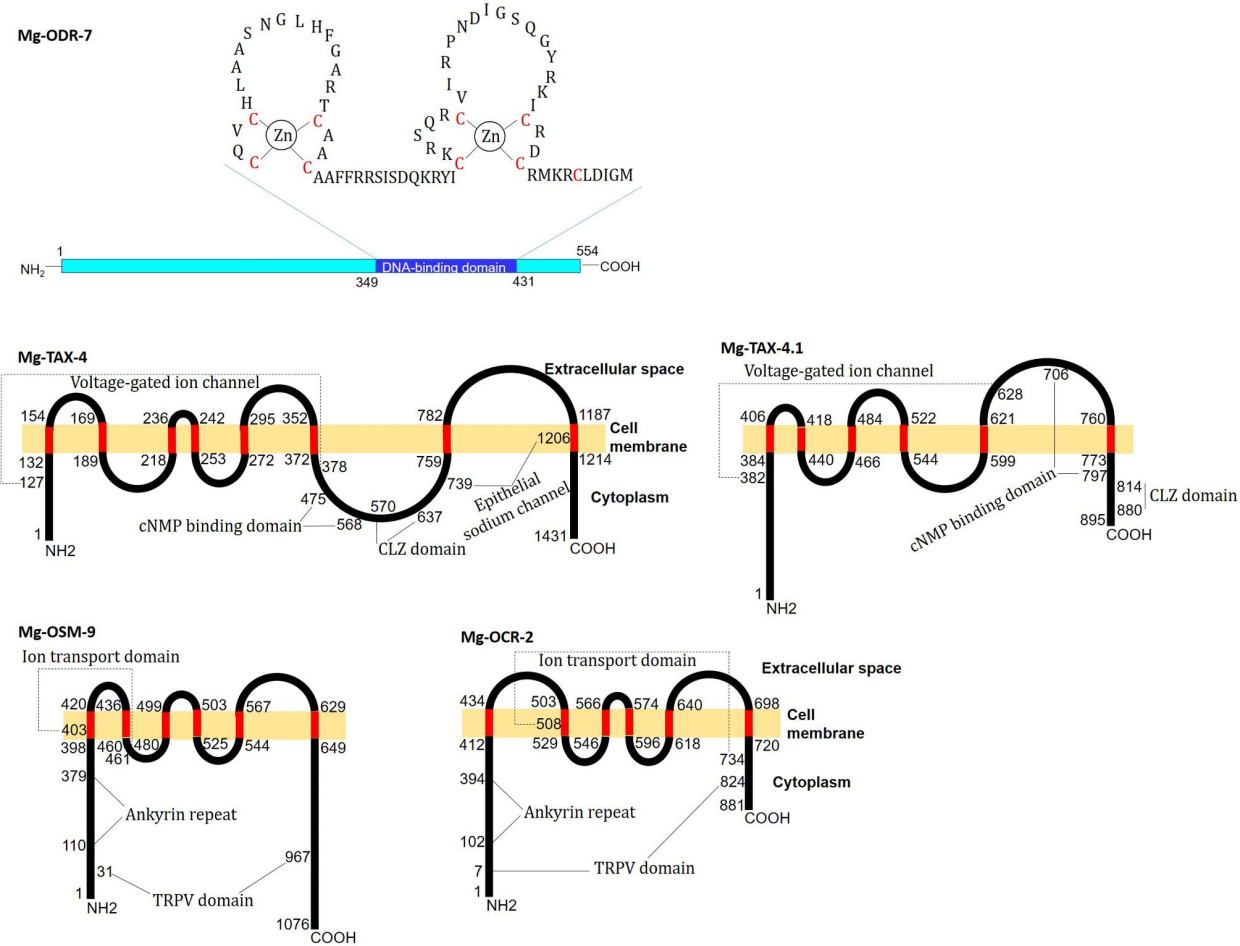
The predicted secondary structures of *M. graminicola* chemosensory gene encoded proteins. Mg-ODR-7 is a cytosol-localized nuclear hormone receptor protein containing a C-terminal DNA-binding domain (349–431 aa) that harbors two Cys4 zinc fingers (nine conserved cysteine residues are shown in red). Mg-TAX-4 contains eight transmembrane motifs (133–153, 170–188, 219–235, 243–252, 273–294, 353–371, 760–781, 1188–1213 aa), ion channel domain (127–378 aa), cytosolic cNMP-binding (475–568 aa) and CLZ (570–637 aa) domains, and a C-terminal epithelial sodium channel (739–1206 aa). Mg-TAX-4.1 contains six transmembrane motifs (385–405, 419–439, 467–483, 523–543, 600–620, 761–772 aa), an ion channel domain (382–628 aa), and predominantly extracellular cNMP-binding (706–797 aa) and cytosolic CLZ (814–880 aa) domains. Mg-OSM-9 contains six transmembrane motifs (399–419, 437–459, 481–498, 504–524, 545–566, 630–648 aa), the characteristic TRPV domain (31–967 aa) that constitutes an ion transport domain (403–461 aa) and N-terminal cytosolic ankyrin repeats (110–379 aa). Mg-OCR-2 contains six transmembrane motifs (413–433, 504–528, 547–565, 575–595, 619–639, 699–719 aa), the characteristic TRPV domain (7-824 aa) that constitutes an ion transport domain (508–734 aa) and N-terminal cytosolic ankyrin repeats (102–394 aa)

The Predicted Mg-ODR-7 sequence is a cytosol-localized nuclear hormone receptor (NHR) protein that contains a C-terminal DNA-binding domain (349–431 aa) that harbours two Cys4 zinc fingers (Fig. 1). Mg-TAX-4 contains eight transmembrane motifs (133–153, 170–188, 219–235, 243–252, 273–294, 353–371, 760–781, 1188–1213 aa), a voltage-gated potassium channel (127–378 aa), a cyclic nucleotide (cAMP/cGMP)-binding domain (475–568 aa), a C-terminal leucine zipper (CLZ) domain (570–637 aa), and an epithelial sodium channel (739–1206 aa) (Fig. 1). Similarly, Mg-TAX-4.1 contains six transmembrane motifs (385–405, 419–439, 467–483, 523–543, 600–620, 761–772 aa), a voltage-gated potassium channel (382–628 aa), cNMP-binding (706–797 aa), and CLZ (814–880 aa) domains (Fig. 1). Mg-OSM-9 contains six transmembrane motifs (399–419, 437–459, 481–498, 504–524, 545–566, 630–648 aa), the exemplary transient receptor potential cation channel subfamily V (TRPV) domain (31–967 aa) that constitutes ankyrin repeats (110–379 aa) and an ion transport domain (403–461 aa) (Fig. 1). Similarly, Mg-OCR-2 contains six transmembrane motifs (413–433, 504–528, 547–565, 575–595, 619–639, 699–719 aa) and a TRPV domain (7-824 aa) comprising ankyrin repeats (102–394 aa) and an ion transport domain (Fig. 1).

Pairwise sequence alignment indicated that the corresponding chemosensory gene sequences are quite shorter in *C. elegans* than those of *M. graminicola* (Supplementary Fig. 1). Although overall sequence identity was merely 31.90% between ODR-7 of *M. graminicola* and *C. elegans*, zinc finger containing DNA-binding domains were quite similar to each other (sequence identity – 71.60%). Mg-TAX-4 and Mg-TAX-4.1 exhibited 44.07 and 60.26% sequence identity, respectively, to *C. elegans* TAX4. Despite lacking the epithelial sodium channel domain, *C. elegans* TAX4 was quite identical to Mg-TAX-4 in terms of voltage-gated ion channel, CNMP-binding, and CLZ domain locations. Mg-OSM-9 and Mg-OCR-2 exhibited 57.47 and 50.90% sequence identity to *C. elegans* OSM-9 and OCR-2, respectively (Supplementary Fig. 1).

The sequence conservation of *M. graminicola* chemosensory genes across the different nematode species was analyzed. Mg-ODR-7, Mg-TAX-4, Mg-TAX-4.1, Mg-OSM-9, and Mg-OCR-2 homologous sequences (belonging to plant-parasitic, animal-parasitic, and free-living nematodes) were retrieved from WormBase Parasite and NCBI non-redundant databases using the BLASTp algorithm. Highly homologous (Percent identity: 50–90%, Query coverage: 50–100%, Expect value: 0.0) sequences were used for the Maximum Likelihood method-based phylogenetic analyses (sequences were additionally annotated to verify the presence of different functional domains). The dendrogram was rooted against the chemosensory gene orthologue of *Drosophila melanogaster* (served as an outgroup). ODR-7 of *M. gramincola* clustered with other PPNs, including root-knot and cyst nematodes (belong to clade 12 of the phylum Nematoda), and the PPN clade branched away from ODR-7 sequences of fungivorous, animal-parasitic, and free-living nematodes (Supplementary Fig. 2), indicating the higher degree of ODR-7 sequence conservation in PPNs. Expectedly, the fungivorous and occasional plant parasites (*Aphelenchoides* spp., *Bursaphelenchus* spp.; clade 10) branched nearer to the PPN clade. A similar result was obtained with TAX-4, TAX-4.1, OSM-9, and OCR-2 sequences (Supplementary Figs. 3–6). However, entries from animal-parasitic and free-living nematodes did not always form identifiable discrete clades, suggesting that these chemosensory genes (especially ODR-7, OSM-9, and OCR-2) are evolutionarily less conserved in nematodes other than PPNs. Overall, a pan-phylum presence of chemosensory genes was documented because ODR-7, TAX-4, TAX-4.1, OSM-9, and OCR-2 homologs were represented in 49, 50, 55, 62, and 55 species of Phylum Nematoda (spanning clades 2, 8, 9, 10, 11, and 12), respectively (Supplementary Table 3). Despite belonging to the identical family of proteins, TAX-4 and TAX-4.1 differed in their representation across the Phylum Nematoda. The same was documented in the case of OSM-9 and OCR-2 proteins (Supplementary Table 3).

### Chemosensory genes are predominantly expressed in the early stage of *M. graminicola*

**Fig. 2:**
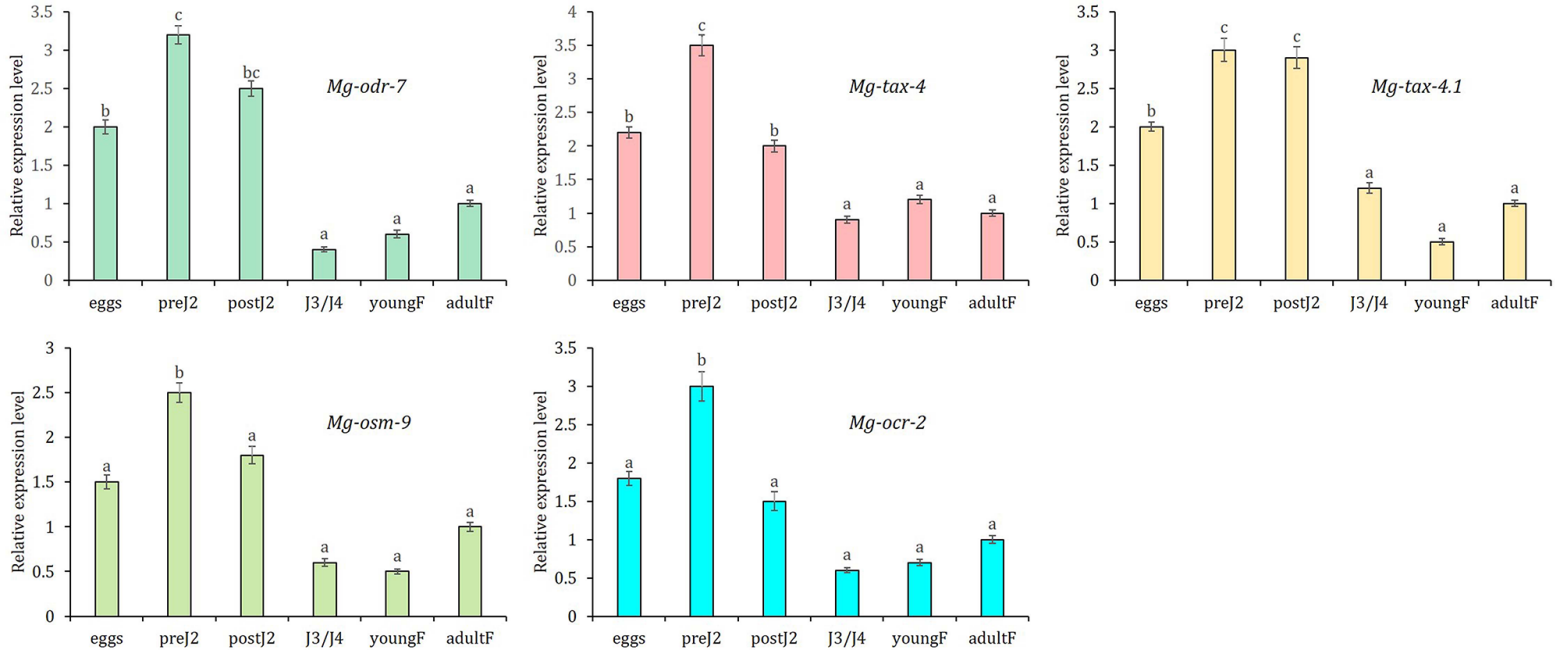
Relative expression level of *Mg-odr-7*, *Mg-tax-4*, *Mg-tax-4.1*, *Mg-osm-9* and *Mg-ocr-2* mRNAs in different developmental stages of *M. graminicola*. Fold change in target gene expression was set as 1 in adult female, and statistically compared with expression in other developmental stages including egg, pre- and post-parasitic J2, J3/J4, young and adult egg-laying female. Each bar represents mean fold change value of qPCR runs in three biological and technical replicates ± standard errors. Bars with different letters indicate significant difference according to the Tukey’s HSD test, *P* < 0.01. Gene expression was normalized using two housekeeping genes of *M. graminicola* (actin and *18 S rRNA*).

RT-qPCR was conducted to examine the variable abundance of *Mg-odr-7*, *Mg-tax-4*, *Mg-tax-4.1*, *Mg-osm-9*, and *Mg-ocr-2* mRNAs across the life stages of *M. graminicola*. The fold change in gene expression was set to 1 in adult females and compared with the fold-change values in other stages. Significantly (*P* < 0.01), the greatest upregulation of *Mg-odr-7*, *Mg-tax-4*, *Mg-osm-9*, and *Mg-ocr-2* was observed in pre-parasitic J2, followed by post-parasitic J2 and egg stage, and the lowest expression was documented in J3/J4 mixed stages and young females (Fig. 2). The greatest (*P* < 0.01) expression of *Mg-tax-4.1* was documented in both pre- and post-parasitic J2 stages, followed by the egg stage (Fig. 2).

### RNAi soaking of *M. graminicola* J2s caused target transcript downregulation

**Fig. 3:**
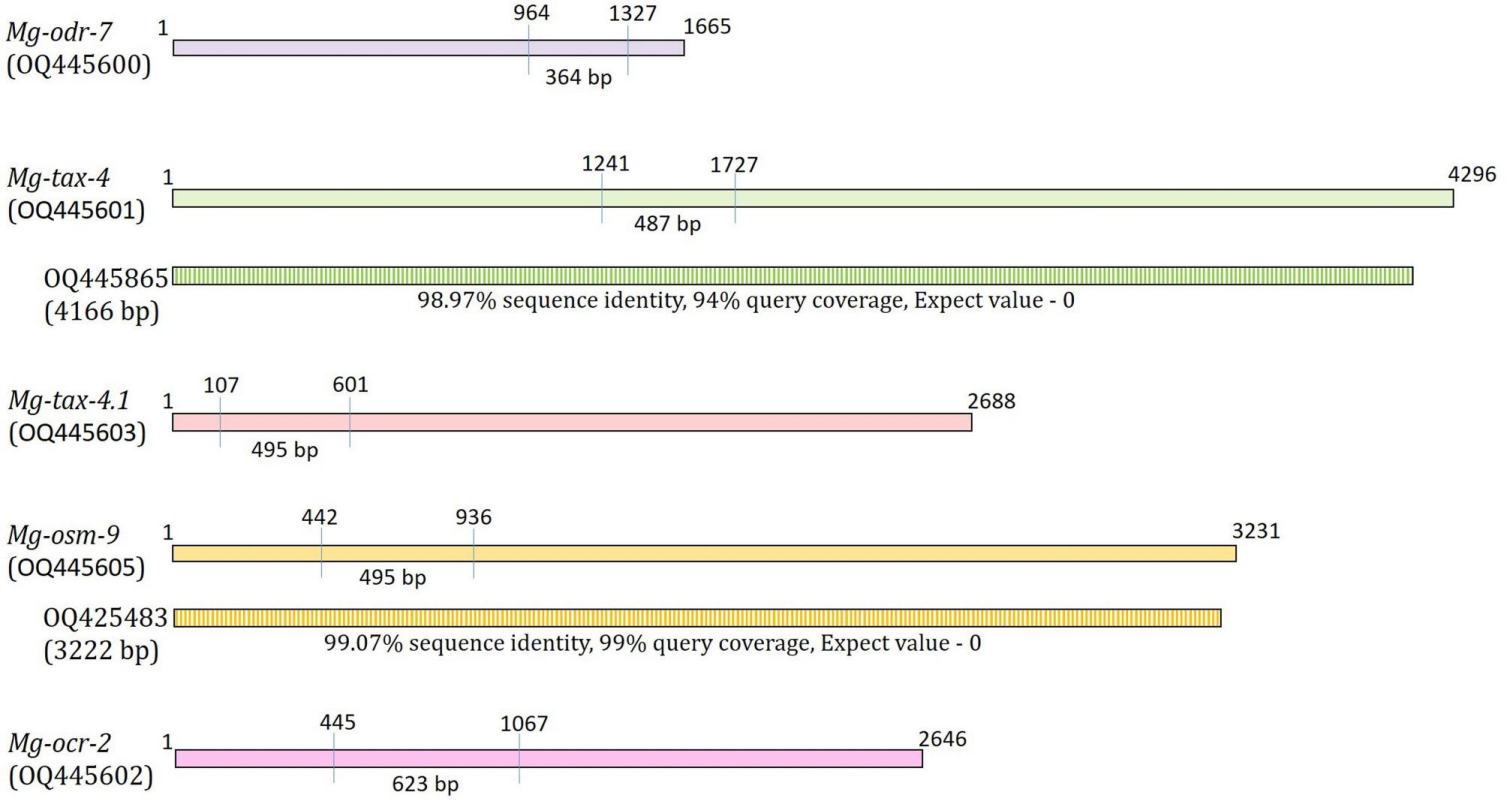
The dsRNA target sites (indicated by perpendicular lines) within the coding sequences of *Mg-odr-7*, *Mg-tax-4*, *Mg-tax-4.1*, *Mg-osm-9* and *Mg-ocr-2* are shown. Numbers indicate the sequence coordinates. The homologous transcripts (indicated by NCBI Genebank identifiers, % identities are also shown) of *Mg-tax-4* and *Mg-osm-9* are provided as oblong shaded boxes

By following two important prerequisites (a. the targeted region should be around 400–600 bp for efficient processing by RNaseIII/Dicer; b. the targeted region should predict greater siRNA formation probability than the non-targeted site of the same transcript), dsRNA molecules were designed from the coding sequences of *Mg-odr-7* (364 bp), *Mg-tax-4* (487 bp), *Mg-tax-4.1* (495 bp), *Mg-osm-9* (495 bp), and *Mg-ocr-2* (623 bp) (Fig. 3). When dsRNA sequences corresponding to *Mg-odr-7*, *Mg-tax-4*, *Mg-tax-4.1*, *Mg-osm-9*, and *Mg-ocr-2* were aligned together, highly discontinuous sequence conservation was observed; despite belonging to an identical family of genes, *Mg-tax-4* and *Mg-tax-4.1* were only 38.7% identical at the nucleotide level (Supplementary Fig. 7). Additionally, to identify probable off-target sequences, our dsRNA sequences were queried against the siRNA databases on the DsCheck (http://dscheck.rnai.jp/) webserver. SiRNAs processed from *Mg-odr-7*, *Mg-tax-2*, *Mg-tax-4*, *Mg-osm-9*, and *Mg-ocr-2* dsRNA did not show a complete match with siRNAs of *C. elegans*, *Drosophila melanogaster*, *Rattus norvegicus*, *Oryza sativa*, and *Arabidopsis thaliana*.

**Fig. 4:**
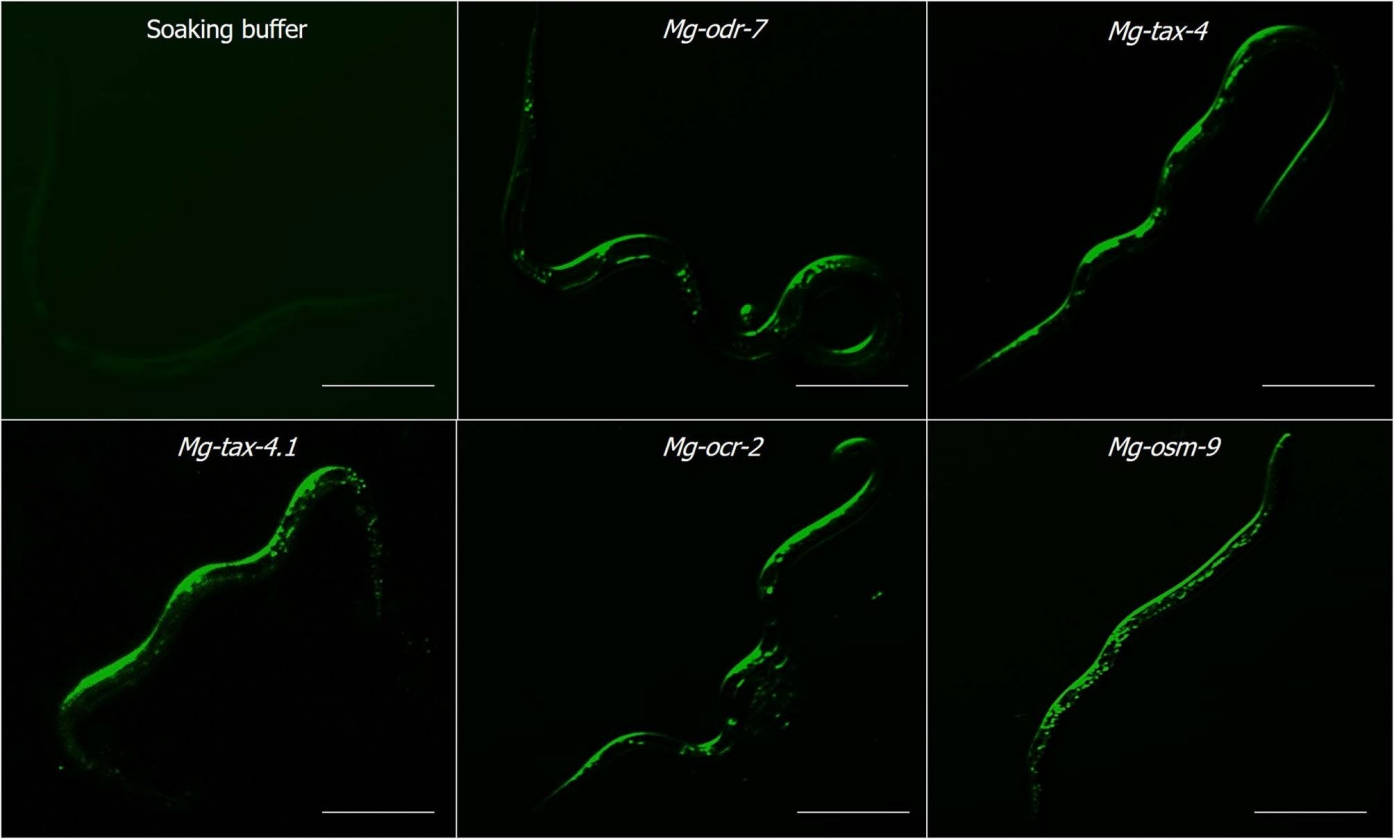
Uptake of dsRNA molecules inside nematode body was visually assessed. DsRNA molecules were labeled with a tracking dye Alexa Fluor 488. Fluorescence imaging showed that dsRNA molecules corresponding to *Mg-odr-7*, *Mg-tax-4*, *Mg-tax-4.1*, *Mg-osm-9* and *Mg-ocr-2* genes were effectively ingested by *M. graminicola* J2 after overnight soaking. Soaking buffer treated worms indicated no fluorescence (slight greenish appearance maybe because of the autofluorescence). Scale bar = 50 μm

J2s were soaked overnight in dsRNA (generated via in vitro transcription) soaking solution. Effective ingestion of dsRNAs (labelled with Alexa Fluor 488 dye) corresponding to all the target genes into the nematode body was confirmed by fluorescence microscopy (Fig. 4).

**Fig. 5:**
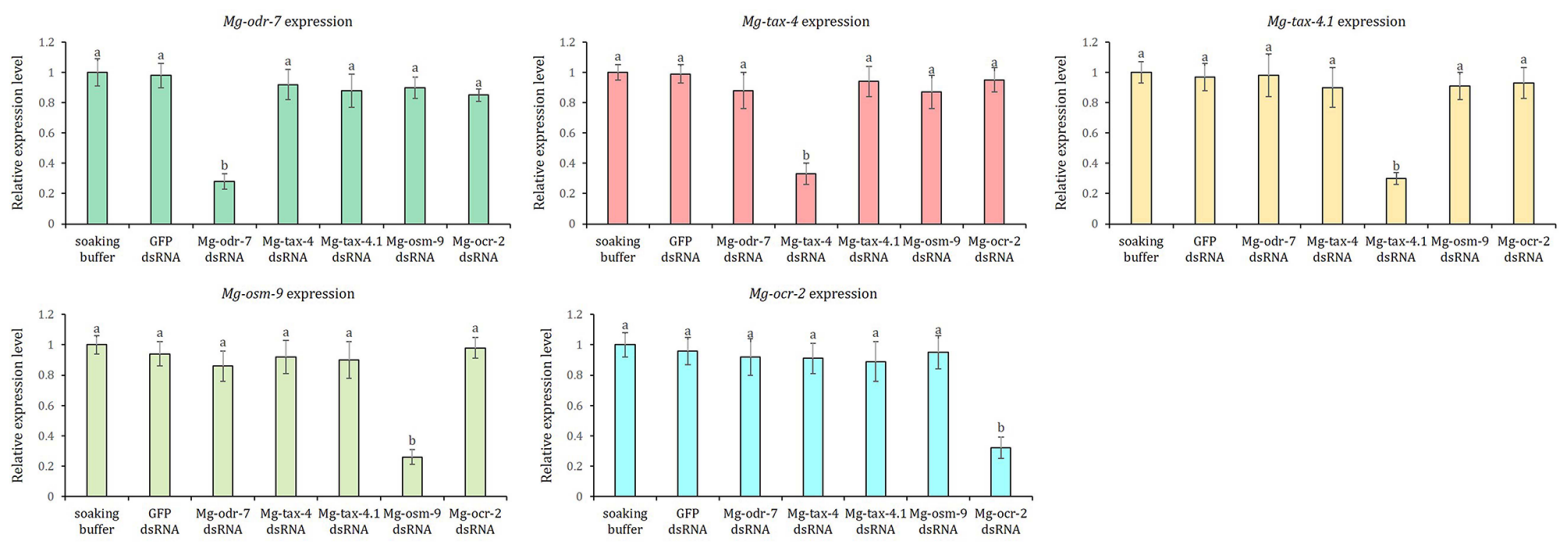
Target-specific downregulation of *Mg-odr-7*, *Mg-tax-4*, *Mg-tax-4.1*, *Mg-osm-9* and *Mg-ocr-2* mRNAs in J2s treated with corresponding dsRNAs at 24 h post soaking. Nematodes treated with *gfp* dsRNA and soaking buffer were used as the non-native and negative control, respectively. *M. graminicola* actin and *18 S rRNA* genes were used to normalize the fold change in expression data. Each bar represents mean fold change value of qPCR runs in three biological and technical replicates ± standard errors. Fold change in target gene expression was set as 1 in soaking buffer-treated worms, and statistically compared with other treatments. Bars with different letters indicate significant difference according to the Tukey’s HSD test, *P* < 0.01

Compared to soaking buffer-treated J2s, 72, 67, 70, 74, and 68% downregulation (*P* < 0.01) of *Mg-odr-7*, *Mg-tax-4*, *Mg-tax-4.1*, *Mg-osm-9*, and *Mg-ocr-2* genes (as determined by qPCR-based gene expression fold change values) were recorded in *Mg-odr-7*, *Mg-tax-4*, *Mg-tax-4.1*, *Mg-osm-9*, and *Mg-ocr-2* dsRNA-soaked J2s, respectively (Fig. 5). Expression of chemosensory genes did not vary (*P* > 0.01) between soaking buffer-treated J2s and *gfp* dsRNA-treated J2s (Fig. 5), exemplifying that dsGFP itself did not cause any negative effect on chemosensory gene transcription. Transcription of *Mg-odr-7* was unaltered (*P* > 0.01) in *Mg-tax-4*, *Mg-tax-4.1*, *Mg-osm-9*, and *Mg-ocr-2* dsRNA-treated J2s and vice versa (Fig. 5). This establishes the target-specific knockdown of olfactory genes in independent RNAi experiments. Nevertheless, *Mg-tax-4* and *Mg-osm-9* shared 98.97 and 99.07% nucleotide sequence identity with their homologous transcripts, OQ445865 and OQ425483, respectively (Fig. 3). It was likely that RNAi of *Mg-tax-4* and *Mg-osm-9* had silenced their other allelic variants in *M. graminicola* J2. It could not be experimentally validated owing to the lack of primer design modules that would distinguish these highly homologous transcripts.

### RNAi of chemosensory genes affected normal behavioral phenotypes of *M. graminicola*

**Fig. 6:**
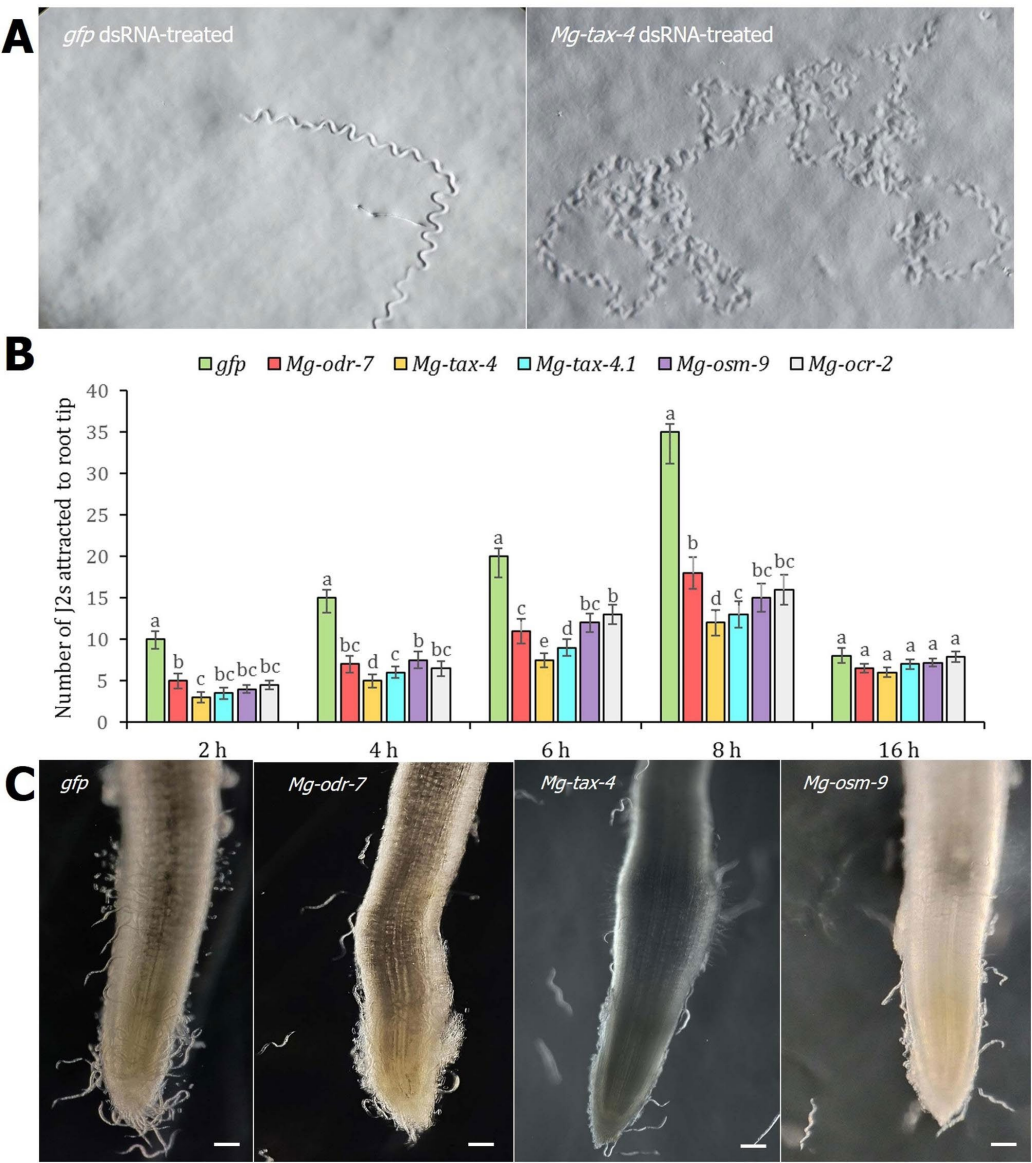
RNAi knockdown of chemosensory genes affected *M. graminicola* host finding ability to rice root. **(A)** J2s were inoculated at 1.5 cm distance behind the root tip and tracks inscribed by J2s in Pluronic gel while finding host were documented. At 2 h after inoculation, a directed proprioception (normal sinusoidal movement) of *gfp* dsRNA-treated J2s were observed in contrast to non-directional proprioception (recurrent changes in direction of movement) of *Mg-tax-4* dsRNA-treated J2s. **(B)** Comparative attraction of dsRNA-treated and control J2s towards root tip at 2, 4, 6, 8 and 16 h after inoculation. Each bar indicates average number of J2s attracted in three biological and five technical replicates ± SE. Bars with different letters are significantly different as per the Tukey’s test, *P* < 0.01. **(C)** Representative images demonstrate lower attraction of *Mg-odr-7*, *Mg-tax-4* and *Mg-osm-9* silenced J2s than control worms at 8 h post inoculation. Scale bar = 100 μm

The proprioception behaviour of dsRNA-treated J2s varied greatly compared to the dsGFP-treated J2s when nematodes were inoculated at 1.5 cm behind the root tip of rice in a Petri dish containing Pluronic gel. Tracks inscribed by control J2s (*n* = 20) exhibited directed sinusoidal locomotion towards the attractant source. On the contrary, tracks inscribed by RNAi worms (*n* = 20) exhibited random and non-directional movement even after 2 h of exposure to the attractant source. A representative photomicrograph is shown in Fig. 6A. Accordingly, *Mg-odr-7*, *Mg-tax-4*, *Mg-tax-4.1*, *Mg-osm-9*, and *Mg-ocr-2* dsRNA-soaked worms were attracted to rice root tips in significantly (*P* < 0.01) lesser numbers than the control J2s at 2, 4, 6, and 8 h (Fig. 6B). At 16 h post inoculation (hpi), no significant difference (*P* > 0.01) in attraction behaviour between RNAi and control J2s was observed (Fig. 6B**)**, possibly because the majority of the attracted worms had invaded the host root by that time. A representative photomicrograph of comparative nematode attraction at 8 h after inoculation is shown in Fig. 6C.

**Fig. 7:**
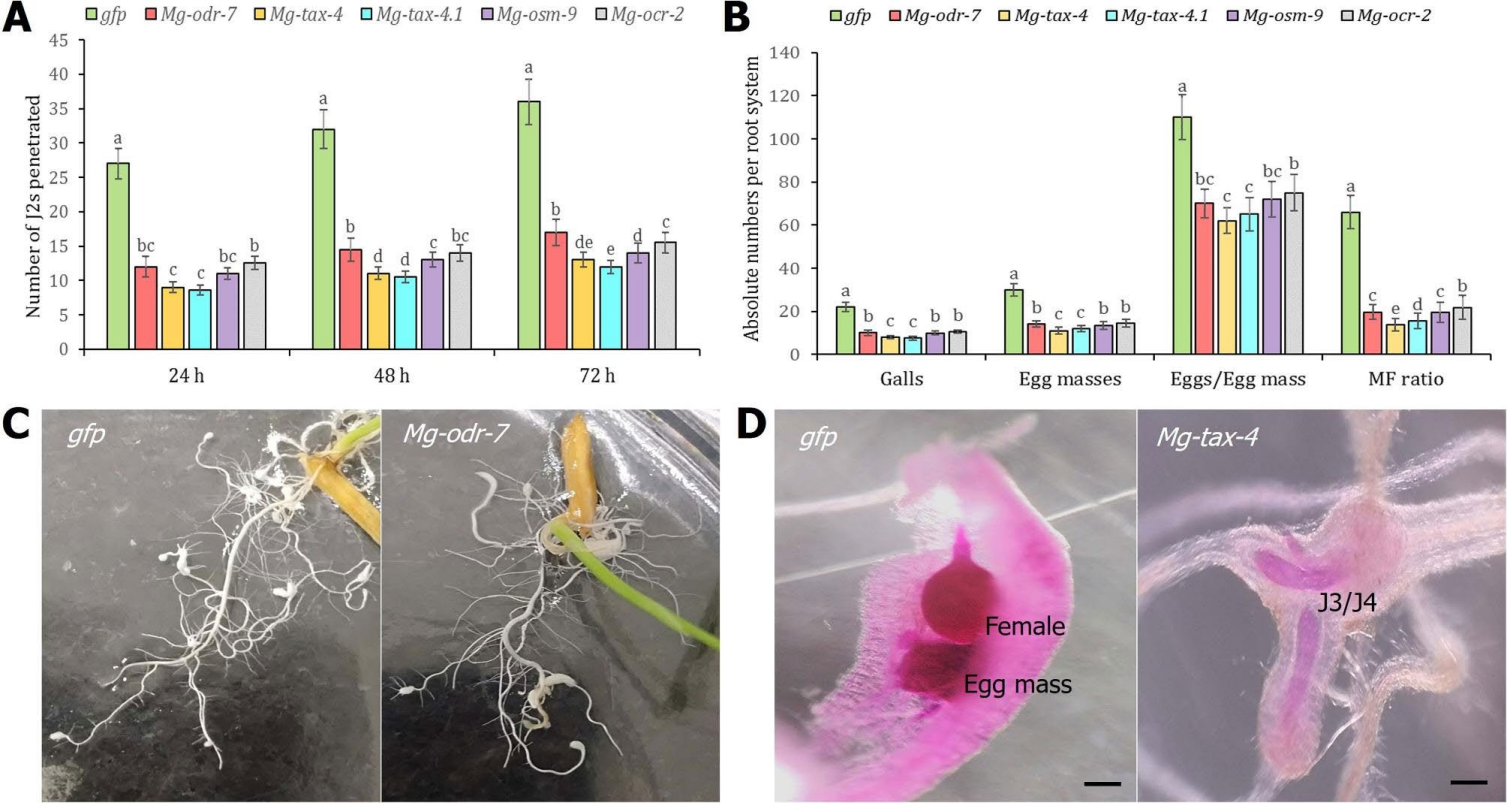
RNAi knockdown of chemosensory genes affected *M. graminicola* parasitic ability in rice root. **(A)** Comparative penetration of dsRNA-treated and control J2s inside the root at 24, 48 and 72 h after inoculation. Roots were stained with acid fuchsin to determine the nematode numbers. Each bar indicates average number of J2s penetrated in three biological and five technical replicates ± SE. Bars with different letters are significantly different as per the Tukey’s test, *P* < 0.01. **(B)** Comparative numbers of galls, egg masses, eggs per egg mass and multiplication factor (MF) ratio of dsRNA-treated and control J2s inside the root at 16 days post inoculation. Each bar indicates average number of different infection parameters in three biological and five technical replicates ± SE. Bars with different letters are significantly different as per the Tukey’s test, *P* < 0.01. **(C)** Representative images demonstrate lower galling intensity in roots infected with *Mg-odr-7* silenced J2s compared to higher galling intensity in roots infected with *gfp* dsRNA-treated J2s. **(D)** Developmental delay was observed in chemosensory gene silenced worms. When control J2s developed to adult females, *Mg-tax-4* silenced J2s developed to J3/J4 mixed stages. Scale bar = 100 μm

Next, roots were stained with acid fuchsin to determine the host penetration potential of RNAi worms. As expected, *Mg-odr-7*, *Mg-tax-4*, *Mg-tax-4.1*, *Mg-osm-9*, and *Mg-ocr-2* dsRNA-soaked J2s penetrated the rice root tip in significantly (*P* < 0.01) lesser numbers than the control J2s at 24, 48, and 72 hpi (Fig. 7A). Consequently, nematode multiplication ability inside host root was affected for RNAi worms than the control worms at 16 days post inoculation (dpi). The numbers of gall, egg mass, eggs per egg mass, and MF ratio per root system were considerably (*P* < 0.01) reduced in roots infected with *Mg-odr-7*, *Mg-tax-4*, *Mg-tax-4.1*, *Mg-osm-9*, and *Mg-ocr-2* dsRNA-soaked J2s compared to the roots infected with control J2s (Fig. 7B, C). A representative photomicrograph shows the developmental delay in RNAi worms than in dsGFP worms (Fig. 7D).

**Fig. 8:**
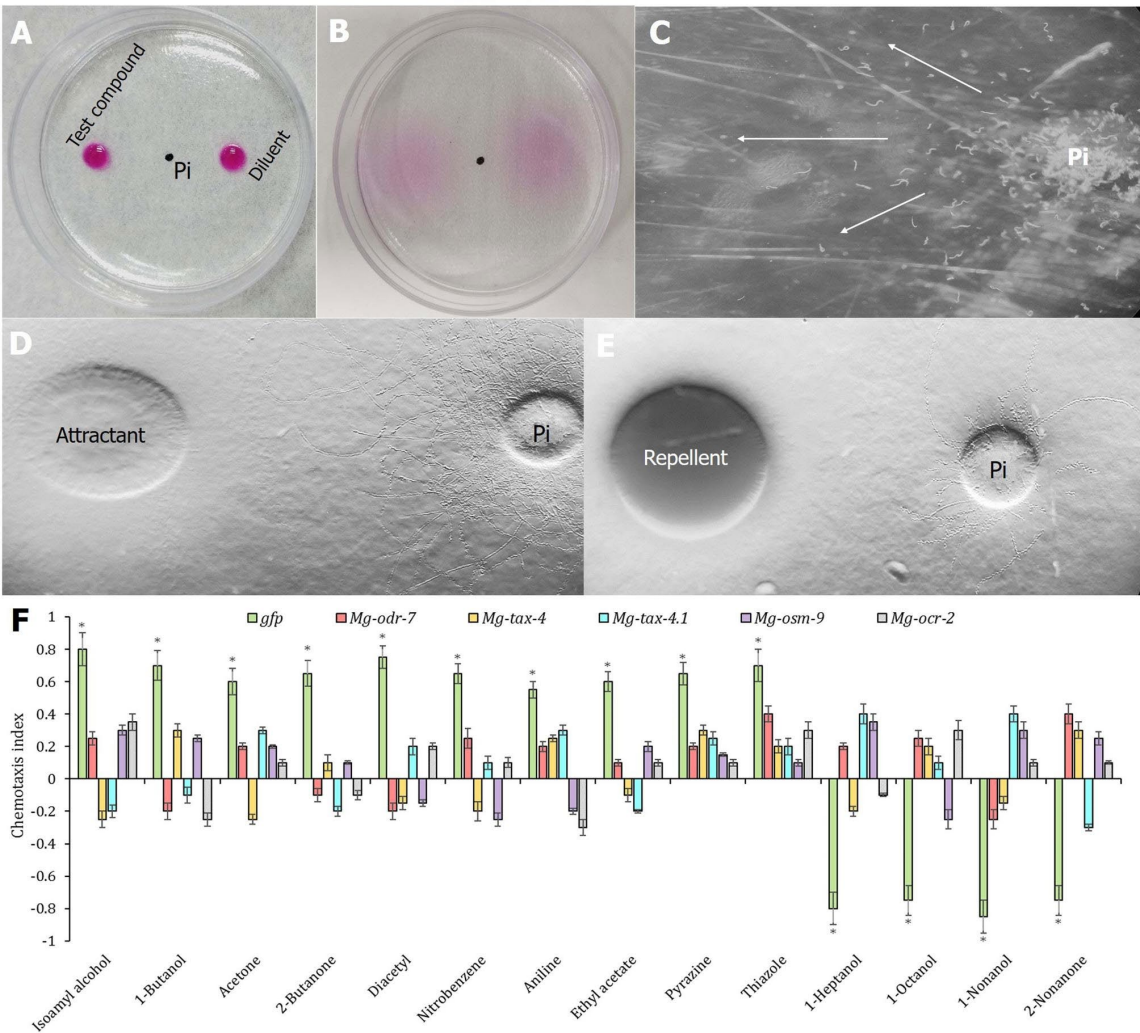
RNAi knockdown of chemosensory genes affected *M. graminicola* chemosensation to volatile organic compounds. In chemotaxis assay, J2s inoculated at the center of a Petri plate containing Pluronic gel. J2 inoculation point is 1.5 cm equidistant from test chemical and its diluent application points. **(A)** Visual assessment of chemical gradient establishment in the assay plate. 10 µL acid fuchsin dye was pipetted to the chemical/diluent application points. **(B)** By 40 min, dyes from chemical/diluent sides slowly dispersed to the inoculation point. **(C)** Photomicrograph depicts selective dispersion of J2s to test chemical side from the inoculation point. **(D)** Numerous tracks visible towards the test chemical side (isoamyl alcohol used here) from the inoculation point of *gfp* dsRNA-treated J2s indicating attraction response. **(E)** Tracks inscribed away from the test chemical side (1-heptanol used here) indicating aversion response. **(F)** Comparative chemotaxis indices (CI, ranges from − 1.0 to + 1.0) of *gfp* dsRNA-treated J2s and *Mg-odr-7*/*Mg-tax-4/Mg-tax-4.1/Mg-osm-9/Mg-ocr-2* silenced J2s towards selective volatile compounds. Each bar indicates average CI from three biological and technical replicates ± SE. Asterisks denote significant difference (*P* < 0.01, paired *t*-test) in CI of J2s towards test chemicals when compared to the CI of J2s towards water (negative control)

Apart from assessing the host finding ability, any perturbation in nematode chemotaxis behavior (towards a broader range of test compounds) due to RNAi knockdown of chemosensory genes was also assessed. In the assay plate, acid fuchsin dye readily dispersed from the application point to establish a concentration gradient and reached the nematode inoculation point by 40 min (Fig. 8A, B). The chemical gradient remained stable for 4 h after application (Supplementary Fig. 8). We assumed that, similar to acid fuchsin, the test compounds may maintain a similar concentration gradient in this assay plate. After 40 min of test compound application, 100 J2s were inoculated at the centre of the plate. Representative photomicrographs show the selective dispersion of J2s towards attractant source or selective aversion from repellent source with reference to the inoculation point (Fig. 8C, D, E).

Initially, the chemotaxis responses of control and RNAi J2s were observed against different volatile organic compounds (sterile ethanol at 0.05% v/v was used as the diluent). Out of six tested concentrations (10^0^, 10^− 1^, 10^− 2^, 10^− 3^, 10^− 4^, and 10^− 5^), the 1% or 10^− 2^ concentration was ultimately used to assess the chemotaxis index of nematodes against volatiles because at this concentration a visible change in the response of wild-type worms was observed. Control J2s exhibited positive chemoreception (*P* < 0.01) or attraction to specific volatiles, including isoamyl alcohol, 1-butanol, acetone, 2-butanone, diacetyl, nitrobenzene, aniline, ethyl acetate, pyrazine, and thiazole, and negative chemoreception (*P* < 0.01) or aversion to specific volatiles such as 1-heptanol, 1-octanol, 1-nonanol, and 2-nonanone (Fig. 8F). By contrast, *Mg-odr-7*, *Mg-tax-4*, *Mg-tax-4.1*, *Mg-osm-9*, and *Mg-ocr-2* dsRNA-soaked J2s did not exhibit any significant change (*P* > 0.01) in chemotaxis response against these attractant and repellent compounds (Fig. 8F).

**Fig. 9:**
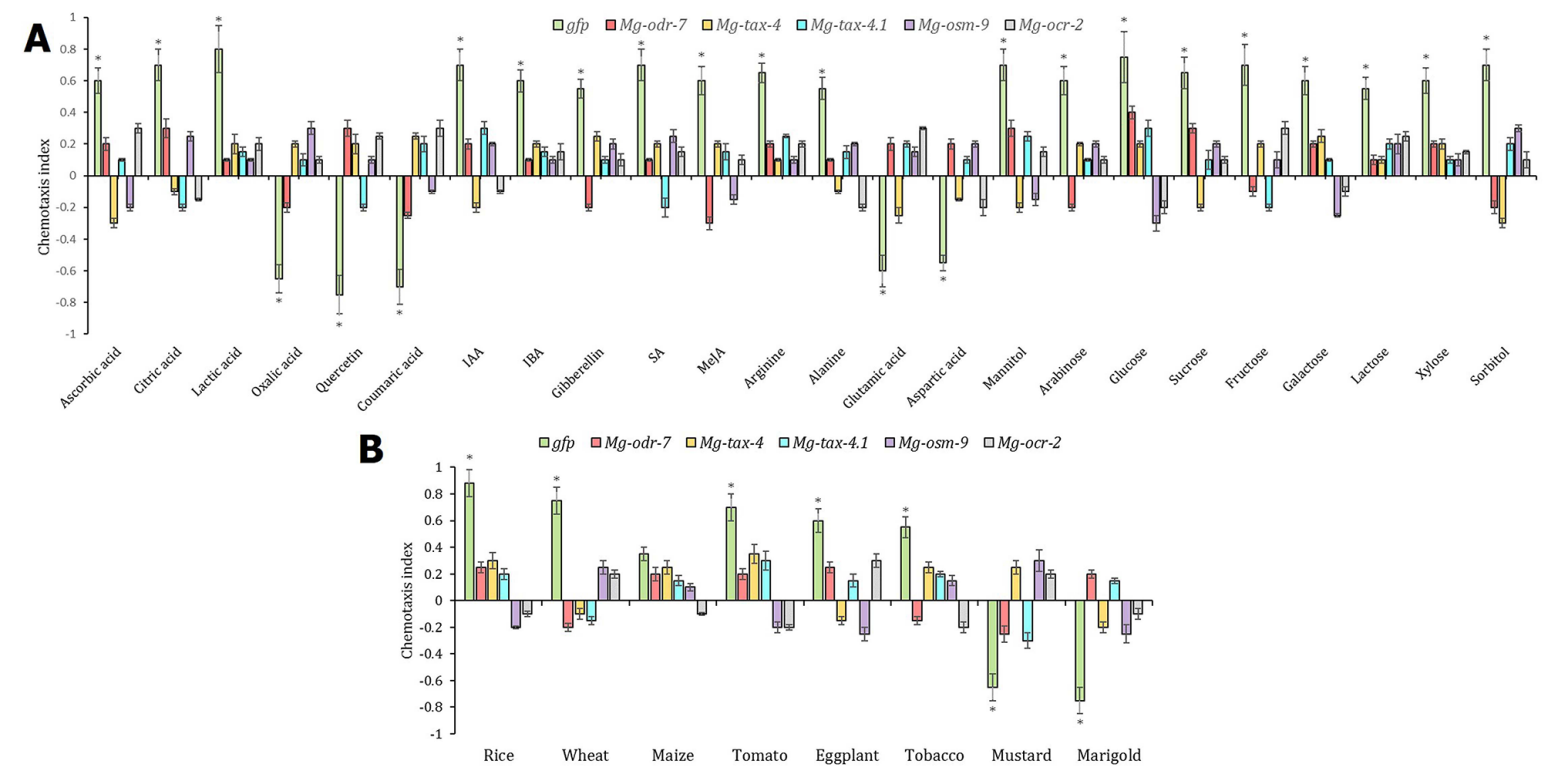
RNAi knockdown of chemosensory genes affected *M. graminicola* chemosensation to nonvolatile organic compounds and plant root exudates. Comparative CI of *gfp* dsRNA-treated J2s and *Mg-odr-7*/*Mg-tax-4/Mg-tax-4.1/Mg-osm-9/Mg-ocr-2* silenced J2s towards selective nonvolatiles (A) and root exudates (B) are shown. Each bar indicates average CI from three biological and technical replicates ± SE. Asterisks denote significant difference (*P* < 0.01, paired *t*-test) in CI of J2s towards test chemicals when compared to the CI of J2s towards water (negative control)

When exposed to different nonvolatile compounds, including organic acids, phenolics, phytohormones (at 200 µM concentration), amino acids, and carbohydrates (at 5 µM concentration), dsGFP J2s exhibited an attraction response (*P* < 0.01) to ascorbic acid, citric acid, lactic acid, indole-3-acetic acid (IAA), indole-3-butyric acid (IBA), gibberellin, salicylic acid (SA), methyl jasmonate (MeJA), arginine, alanine, mannitol, arabinose, glucose, sucrose, fructose, galactose, lactose, xylose, and sorbitol, and an aversion response (*P* < 0.01) to oxalic acid, quercetin, coumaric acid, glutamic acid and aspartic acid (Fig. 9A). On the contrary, RNAi J2s did not show any significant change (*P* > 0.01) in their chemotaxis response towards these attractant and repellent compounds (Fig. 9A). Upon exposure to different host root exudates, dsGFP J2s exhibited an attraction response (*P* < 0.01) to rice, wheat, tomato, eggplant, and tobacco exudates and an aversion response (*P* < 0.01) to mustard and marigold exudates (Fig. 9B). However, RNAi J2s did not show any significant change (*P* > 0.01) in their chemotaxis response towards these attractant and repellent root exudates (Fig. 9B). Notably, neither of the control or RNAi J2s exhibited attraction or repulsion (*P* > 0.01) to maize root exudates (Fig. 9B).

## Discussion

Perturbation of PPN sensory perception towards host roots is considered a novel and environmentally sustainable nematode management strategy [13–15]. However, compared to the vast literature on *C. elegans* chemosensory biology [18], negligible progress has been made in characterizing chemosensory genes from PPNs. The current study advances that knowledge by identifying and deciphering the functional roles of five chemosensory genes (*Mg-odr-7*, *Mg-tax-4*, *Mg-tax-4.1*, *Mg-osm-9*, and *Mg-ocr-2*) from *M. graminicola*. Downstream of GPCRs, two major signal transduction channels, i.e., cyclic nucleotide-gated (CNG) and transient receptor potential subfamily V (TRPV) channels, mediate sensory perception in *C. elegans* [18, 24]. Upon binding of cGMP/cAMP to its intracellular cNMP-binding domain, the CNG channel generates cation fluxes, which eventually lead to olfactory signal amplification [25, 26]. Tetrameric CNG channels constitute 1–4 α subunits (TAX-4) and differing numbers of β subunits (TAX-2) arranged around a central pore [25, 26]. Mutations in *tax-2*/*tax-4* led to defective chemotaxis in *C. elegans* [17, 18]. Similarly, mutations in TRPV family members such as *osm-9*/*ocr-2* attenuated the cation fluxes that ultimately led to perturbed chemosensation in *C. elegans* [17, 18]. Opening of CNG and TRPV channels results in the attractive response of *C. elegans* [19]. Additionally, ODR-7, a member of the large divergent nuclear receptor family, regulates the expression of different chemosensory genes; mutations in *odr-7* affect the odorant response in *C. elegans* [27, 28].

In this study, we report the full-length cDNA sequence of *Mg-odr-7*, which encodes a 554 aa long Mg-ODR-7 protein containing two Cys4 zinc fingers in its DNA-binding domain. A typical nuclear receptor (which acts like a transcription factor that regulates the expression of genes) contains a DNA-binding domain (two zinc fingers bind to DNA, ligand-independent transcriptional activation function) followed by a ligand-binding domain (which recruits coactivators or corepressors, ligand-dependent function) [29]. However, alike of *C. elegans* ODR-7 [28], Mg-ODR-7 lacks the ligand-binding domain, and the DNA-binding domain is displaced to the carboxyl terminus.

In addition, we report the full-length cDNA sequences of different ion channel proteins. Mg-TAX-4 (1431 aa) contains eight transmembrane motifs, the typical cNMP-binding domain, a voltage-gated potassium channel, an epithelial sodium channel, and a C-terminal leucine zipper. We identified a homologue of Mg-TAX-4, named Mg-TAX-4.1 (895 aa). Mg-TAX-4.1 contains six transmembrane motifs, a cNMP-binding domain, a voltage-gated potassium channel, and a C-terminal leucine zipper, but lacks the epithelial sodium channel. A pairwise comparison showed that Mg-TAX-4 and Mg-TAX-4.1 are not very similar to each other (amino acid sequence identity – 49.51%), suggesting their identity as different genes. In our earlier study in *M. incognita*, we named the *Mi-tax-4* homologous transcript *Mi-tax-2*, indicating their ambiguous nomenclature [9]. Notably, in *C. elegans*, the major difference between TAX-4 and TAX-2 is the presence and absence of the C-terminal leucine zipper domain, respectively [30, 31]. This domain is extremely crucial because it regulates the assembly of CNG channel subunits [30].

TRPV channel proteins Mg-OSM-9 (1076 aa) and Mg-OCR-2 (881 aa) contain six transmembrane motifs and the typical TRPV domain that harbors N-terminal ankyrin repeats and an ion transport domain. Apart from their direct role in sensory signal transduction, *C. elegans* OSM-9 and OCR-2 are implicated in regulating the expression of chemosensory genes, possibly because of the presence of ankyrin repeats [32–34]. Findings suggested that a physical interaction between OSM-9 and OCR-2 would be necessary for smooth transduction of chemosensory signals [33].

In our earlier study [9], we analyzed the function of *M. incognita odr-1*, *odr-3* and *tax-2* (*tax-4* reported in that paper was a homologue of *tax-2*, this ambiguity was mentioned in that paper) based on the partial sequences cloned. Herein, we analyzed the function of different family of chemosensory genes from *M. graminicola* such as *odr-7*, *tax-4*, *osm-9* and *ocr-2* based on the full-length sequences cloned. Since full-length sequences of these genes were not available in other PPNs, domain-wise comparison was made with the *C. elegans*.

A pairwise sequence alignment showed that all the chemosensory gene sequences are quite shorter in *C. elegans* than in *M. graminicola*, indicating a putative gene acquisition in PPNs during their functional divergence from free-living ones. Additionally, phylogenetic studies demonstrated that Mg-ODR-7, Mg-TAX-4, Mg-TAX-4.1, Mg-OSM-9, and Mg-OCR-2 sequences are evolutionarily highly conserved in plant-parasites, because PPN sequences clearly branched away from their homologues in animal-parasites and free-living nematodes. This can be attributed to the hypothesis that plant parasites have independently evolved at least four times within the phylum Nematoda [35]. A pan-phylum conservation analysis of chemosensory gene sequences validated the separate identities of Mg-TAX4/Mg-TAX4.1 and Mg-OSM-9/Mg-OCR-2 sequences (based on the presence or absence of homologous sequences in specific nematode species). The greater conservation of candidate gene sequences in PPNs than other feeding groups is useful for developing chemodisruptive drugs or RNAi plants (that express PPN-specific dsRNA or siRNA sequences) that would have no off-target effect on beneficial nematodes.

Our qPCR-based gene expression analysis revealed that *Mg-odr-7*, *Mg-tax-4*, *Mg-tax-4.1*, *Mg-osm-9*, and *Mg-ocr-2* transcripts are most abundantly expressed in the early life stages of *M. graminicola*, particularly in pre-parasitic J2 stages, implicating their putative roles in nematode chemotaxis and host finding processes. To further validate this hypothesis, we resorted to RNAi-based functional analysis of chemosensory genes. J2s were soaked with dsRNA molecules from specific genes, and target transcript downregulation was ascertained. Next, a number of behavioural phenotypic assays were performed. When inoculated away from the host root, dsRNA-treated nematodes exhibited dwelling-type (recurrent local roaming due to inability to find the attractant source) proprioception in contrast to the directed locomotion (global roaming towards the attractant source) in control nematodes. The chemosensory gene-mediated pirouette model of nematode chemotaxis is well established in *C. elegans* [17]. In our earlier study, we reported similar dwelling movements in *M. incognita* J2s when *Mi-odr-1*, *Mi-odr-3*, *Mi-tax-2*, and *Mi-tax-4* genes were silenced via RNAi [9].

Comparative attraction analysis towards the rice root tip showed that dsRNA-treated J2s were attracted in significantly lesser numbers than control J2s at 2, 4, 6, and 8 h post-inoculation. Nevertheless, RNAi and control J2s were attracted alike at 16 h, suggesting the probable transient effect of RNAi [20, 36]. Comparative penetration analysis showed that dsRNA-treated J2s invaded the rice root in significantly lesser numbers than control nematodes at 24, 48, and 72 h. Consequently, the life cycle progression of RNAi worms was considerably affected (developmental delay was also documented) in host roots compared to control worms, as revealed by the number of galls, egg mass, egg per egg mass, and multiplication factor ratio data. The detrimental effect on nematode behavioural phenotypes due to independent downregulation of *Mg-odr-7*, *Mg-tax-4*, *Mg-tax-4.1*, *Mg-osm-9*, and *Mg-ocr-2* suggests that each of these chemosensory genes performs vital functions in the *M. graminicola* chemosensation process. Notably, ODR-7 has a transcriptional activation function [29]. The function of the CNG channel is dependent on the assembly of different TAX subunits in *C. elegans* [25, 26]. Interaction between OSM-9 and OCR-2 is necessary for TRPV channel-mediated chemosensory signal transduction in *C. elegans* [33].

The volatile and nonvolatile organic compounds emanating from the plant root exudates can selectively mediate PPN host-finding behaviour in the rhizosphere [37–40]. Using its olfactory genes, *C. elegans* selectively chemotax to alcohols, esters, amines, ketones, organic acids, aromatic, and heterocyclic compounds [27, 41]. The adaptive chemosensory response (selective discrimination and chemo-orientation to different compounds) towards different organic compounds has been demonstrated in PPNs, including *Meloidogyne* spp. and *Globodera* spp. [9, 42–45]. In view of these findings, we investigated the comparative chemotaxis responses of control and RNAi J2s towards a wide spectrum of volatile and nonvolatile organic compounds and root exudates. Among the volatiles, control J2s exhibited attractive and aversive responses to 10 and 4 compounds, respectively. Among the nonvolatiles, control J2s showed attractive and aversive responses to 19 and 5 compounds, respectively. Among the root exudates, control J2s exhibited variable responses such as attraction (rice, wheat, tomato, eggplant, and tobacco), repulsion (mustard, marigold), and no response (maize). Conversely, RNAi J2s did not show a significant attractive or aversive response to either of these test compounds, suggesting RNAi-induced defective chemotaxis in *M. graminicola*.

In conclusion, the present study describes the vital and independent roles of *Mg-odr-7*, *Mg-tax-4*, *Mg-tax4.1*, *Mg-osm-9*, and *Mg-ocr-2* in *M. graminicola* chemosensation and host location processes. Chemotaxis is the most important part of PPN sensory biology because it modulates key biological processes such as migration in soil, finding a suitable host, invading the host, and initiating a feeding cell inside the host tissue. The molecular basis of PPN chemoreception, or specifically, unravelling the PPN chemosensory mechanism, is yet an underexplored research area. In this direction, extensive research must be undertaken to deorphanize PPN-specific chemosensory receptors and identify the downstream signal transduction pathway.

## Materials and methods

### Plant material and nematode culturing

Seeds of *Oryza sativa* cv. Pusa Basmati 1121 were soaked overnight in sterile distilled water. Next, seeds were surface-sterilized with 70% ethanol for 1 min, washed with sterile water, and placed in a Petri dish containing moist filter paper. Petri dish was incubated at 28 °C and 60% relative humidity (RH) in a growth chamber. Four- to five-day-old germinated seedlings were used in the following experiments.

A pure culture of *M. graminicola* (obtained from the IARI rice field, species identity was confirmed by analyzing the perineal pattern of females and using a species-specific SCAR-PCR molecular marker) was propagated in rice (*O. sativa* cv. Pusa Basmati 1121) roots growing in the pot soil in a green house at 28ºC, 60% RH and 14:10 h light:dark photoperiod. The egg masses were collected from the galled roots (using sterilised tweezers) at 30 dpi and incubated in a Petri dish containing sterile tap water for 24–48 h. Newly-hatched pre-parasitic second-stage juveniles (J2s or preJ2s) were used in the subsequent experiments. For gene expression studies, different developmental stages of *M. graminicola* including post-parasitic J2s (postJ2s, at 3 dpi), J3/J4s (7 dpi), young (12 dpi), and egg-laying adult females (16 dpi), were dissected out of the infected roots using sterilised scalpels and tweezers.

### Collection of root exudates

Root exudates were collected from the hydroponically grown host plants as described previously [8, 38]. Briefly, seeds of different host plants, including rice (cv. Pusa Basmati 1121), wheat (cv. Sonalika), maize (cv. Buland), tomato (cv. Pusa Ruby), eggplant (cv. Pusa Purple Long), tobacco (cv. Petit Havana), mustard (cv. Pusa Jai Kisan), and marigold (cv. Arpit), were surface-sterilized with 70% ethanol and germinated in sterile, moist Whatman No. 1 filter paper disks in Petri plates at 28ºC in dark. Four- to five-day-old seedlings (*n* = 30) of identical host plant were pooled together and their root tips were immersed in 50 mL Falcon tubes (Corning) containing the Hoagland solution. Root exudates were periodically collected from the tube at 2 days interval till 15–20 days and replaced with sterile Hoagland solution. Pooled exudates were filtered through Whatman No. 1 paper and concentrated 50-fold in a vacuum concentrator (Labconco, Biogentek). 10 µL of lyophilized exudates were used as the test compound in chemotaxis bioassays.

### Bioinformatics

Firstly, known amino acid sequences corresponding to *C. elegans odr-7*, *tax-2*, *tax-4*, *osm-9*, and *ocr-2* genes were queried against the translated nucleotide sequence database of *M. graminicola* in WormBase Parasite (https://parasite.wormbase.org/). Retrieved sequences (having top-scoring reciprocal BLAST hits in terms of least expect value and greatest bit score) were additionally validated against the published genomic and transcriptomic resources of *M. graminicola* [46, 47]. Full-length cDNA sequences (amplified using rapid amplification of cDNA ends (RACE)-PCR, described in the subsequent sections) were queried in NCBI CDD (https://www.ncbi.nlm.nih.gov/cdd/), InterProScan (https://www.ebi.ac.uk/interpro/search/sequence/), and FGENESH (http://www.softberry.com/) to verify the characteristic conserved domains in different chemosensory genes. Multiple sequence alignment of candidate genes with their corresponding homologs (encompassing different species from Phylum Nematoda) using the Clustal Omega (https://www.ebi.ac.uk/Tools/msa/clustalo/) tool. The evolutionary relationship of candidate genes with their homologs was interrogated using the MEGA6 phylogeny tool, in which Maximum Likelihood method was adopted and Tamura 3 parameter model was followed. Bootstrap consensus was obtained from 1000 replicates, and phylogenetic tree branches pertaining to < 70% bootstrap replicates were collapsed. A discrete Gamma distribution model was followed to predict evolutionary rate differences across the tree.

### Nematode RNA extraction

Total RNA was isolated from the J2s of *M. graminicola* using the NucleoSpin® RNA kit (Macherey-Nagel) in accordance with the manufacturer’s protocol. The quantity and quality of extracted RNA were examined in a Nanodrop ND-1000 spectrophotometer (Thermo Fisher Scientific) followed by electrophoresing in 1% (w/v) agarose gel. The purified RNA (~ 500 ng) was converted into first-strand cDNA using a reverse transcription (RT)-PCR kit (Superscript VILO, Invitrogen); cDNAs were stored at – 20 °C for downstream applications. For nematode gene expression analyses, RNAs were isolated from different life stages of *M. graminicola* and converted into cDNAs as depicted earlier.

### Full-length cloning of cDNA sequences

The first-strand cDNA of *M. graminicola* J2 was used as the template and primed with an oligo(dT) primer and Smart II A oligonucleotide to generate 5’ and 3’-RACE-ready cDNAs using the RACE cDNA amplification kit (Clontech, TaKaRa) by following the manufacturer’s instructions. 3’- and 5’-RACE fragments were synthesized by priming with sense and antisense gene-specific primers (GSP), respectively, along with universal primers. Amplified products cloned onto the pGEM-T Easy (Promega) vector were Sanger sequenced. Using primer walking, the complete cDNA sequence was obtained. The open reading frame (ORF) of different chemosensory genes was submitted to the NCBI GenBank database. Primer details are given in Supplementary Table 1.

### Gene expression study

A quantitative PCR (qPCR)-based gene expression study was performed in a CFX96 thermal cycler (BioRad). 10 µL of reaction mixture comprised of 1.5 ng first-strand cDNA, 750 nM each of sense and antisense primers, and 5 µL SYBR Green qPCR master-mix (BioRad). The thermal cycling programme included a hot start of 95 °C for 30 s, followed by 40 cycles of 95 °C for 10 s and 60 °C for 30 s. Amplification specificity was determined by adding a melt curve programme (95 °C for 15 s, 60 °C for 15 s, followed by a slow ramp from 60 to 95 °C). Quantification cycle (Cq) values were retrieved from CFX Maestro software (BioRad) and fold change in target gene expression was calculated by the 2^−ΔΔCq^ method. Gene expression was normalized using two housekeeping genes of *M. graminicola*, i.e., *18 S rRNA* and actin. Three biological and technical replicates were included for each sample. Efficiency of primers was calculated by generating a standard curve (Cq values plotted against cDNA concentrations) from a five-fold dilution series of *M. graminicola* cDNA; a linear regression equation (*E* = (10^(−1/slope)^ – 1) × 100) was employed to analyze the slope. Primer details and their reaction efficiency are given in Supplementary Table 2.

### In vitro synthesis of dsRNA molecules

A number of bioinformatics tools, including E-RNAi (https://www.dkfz.de/signaling/e-rnai3/), siDirect (https://sidirect2.rnai.jp/), Dharmacon (https://horizondiscovery.com/) and dsCheck (https://dscheck.rnai.jp/) were used to design dsRNAs from the chemosensory genes. These tools predicted the greatest siRNA formation probabilities in the targeted region of the candidate gene and averted probable off-target effects by interrogating the sequence homologies of putative siRNAs with siRNAs from non-target organisms. DNA sequences of targeted dsRNA regions were PCR-amplified from nematode cDNA and cloned into pGEM-T. Primer details are given in Supplementary Table 2.

Sequences corresponding to candidate dsRNAs were PCR-amplified from recombinant pGEM-T clones using M13 primers. Gel-purified PCR products of each candidate gene were in vitro transcribed to single-strand sense and antisense RNAs in separate reactions using T7 and SP6 RNA polymerase (MEGAscript, Invitrogen), respectively. Sense and antisense RNAs were mixed together and incubated at 65 °C for 10 min, followed by 37 °C for 30 min to synthesize dsRNA molecules of different candidate genes. The generation of dsRNA was ascertained by electrophoresing a 1 µL aliquot in 1% (w/v) agarose gel. In addition, dsRNA of a green fluorescent protein (GFP)-encoding gene was synthesized to be used as the control.

### Soaking of nematodes in dsRNA solution

500 J2s were pre-treated with RNase-free water and soaked overnight in a soaking buffer containing 1 mg mL^− 1^ dsRNA and 50 mM octopamine in an Eppendorf tube placed in a rotator in the dark at 28 °C. In separate experiments, fluorescent dye-labeled dsRNAs (custom synthesized dsRNAs were fused with Alexa Fluor 488 dUTP) were added to the soaking buffer to visually examine the uptake of dsRNA molecules inside the nematode body. Fluorescence imaging (excitation wavelength − 488 nm, emission wavelength − 520 nm) was performed in a Zeiss Axiocam MRm microscope.

After RNAi treatment, J2s were rinsed with RNase-free water for five times for a duration of 2 min each. Total RNA was isolated from J2s and reverse-transcribed to cDNA, as explained earlier. Targeted downregulation of candidate genes was analyzed by qPCR; amplification conditions were the same as explained earlier.

### Assessment of RNAi phenotypes in *M. graminicola*

Migration behaviour of the RNAi worms towards rice root was assessed in a Petri dish (50 × 10 mm) containing Pluronic gel or PF-127 medium (Sigma). PF-127 is a semisolid, transparent medium that allows observing the nematode’s tracking behaviour in real time. 23% gel was prepared by dissolving the 23 g PF-127 powder in 80 mL distilled water [48] in a bottle, which was autoclaved and stored at 4ºC. The root tip of a four- to five-day-old seedling was put at the centre of the plate containing 23% PF-127 medium. Approximately 20 J2s were inoculated at a distance of 1.5 cm from the root tip, and the J2 movement pattern in terms of sinusoidal tracks inscribed on the gel was recorded under the microscope. For obtaining representative images, movement of individual J2s was recorded for 1 h.

Next, the host location ability of RNAi worms towards rice root was assessed in Petri dishes (50 × 10 mm) containing 23% PF-127 medium. 5 mL of gel was poured into the gel and solidified at room temperature. The root tip of a four- to five-day-old seedling was put (by immersing the tip in the gel) at the centre of the plate, and approximately 50 J2s were inoculated at a distance of 1.5 cm from the root tip. Plates were incubated in trays (containing moist filter papers) in a BOD incubator at 28 °C and 60% humidity in dark. The attraction of J2s to the root tip (J2s touching the root tip and at 2 mm area around the root tip, as depicted in Dutta et al. [49] and Dutta and Akhil [50]) was microscopically observed and counted at 2, 4, 6, 8, and 16 h after inoculation. The attraction patterns of control worms are provided in the Supplementary Fig. 9. Additionally, the number of J2s that penetrated the root (stained with acid fuchsin dye by following the method described in Byrd et al. [51]) was counted at 24, 48, and 72 h after inoculation. Comparative penetration potential of control and RNAi worms at 24 h is provided in the Supplementary Fig. 10.

The parasitic success of RNAi worms inside the rice root was also assessed. Fifty J2s were inoculated near the root tip of a four- to five-day-old seedling in larger Petri dishes (110 × 25 mm; HIMEDIA, Code number: PW1147) containing 20 mL of 23% PF-127 medium [52]. Covered Petri plates were placed in a humid tray and kept at 28 °C and 60% humidity in a BOD incubator (Thermotech) with 16:8 h light:dark photoperiod (light level − 200 µmol photons m^− 2^ s^− 1^). A representative image of these plates at 16 days after inoculation is provided in Supplementary Fig. 11. At 16 days, plants were harvested from the gel by placing plates briefly over an ice bath. Gel was liquefied at low temperature and contaminant-free plants could be easily extracted without any damage to the root system. Different infection parameters such as number of galls, number of egg masses (which represent the number of reproducing females because each female produces its progeny in an egg mass), number of eggs per egg mass, and nematode multiplication factor (MF) ratio [(number of egg masses × number of eggs per egg mass) ÷ initial inoculum level] per root system were quantified. At least five biological and three technical replicates were kept for each treatment.

The chemotaxis behaviour of RNAi worms towards various test chemicals (including root exudates and volatile and nonvolatile compounds) was analyzed in a Petri dish (50 × 10 mm) containing 5 mL of 23% PF-127 medium. Test chemicals (10 µL) were pipetted at a distance of 1.5 cm from the centre of the plate. The diluent of the test chemical was pipetted at the opposite end, 1.5 cm from the centre of the dish. After chemical gradient establishment (this was visually assessed by using acid fuchsin dye), approximately 100 J2s were inoculated in the centre of the plate. Dishes were incubated at 28 °C, and at 1 h after J2 inoculation, J2s chemotaxed to the test chemical end and diluent end were documented under the microscope. The experimental setup is schematically represented in Supplementary Fig. 12. Finally, the chemotaxis index (CI) was calculated using the following formula: [(number of J2s chemotaxed to the test chemical end – number of J2s chemotaxed to the diluent end) ÷ total number of J2s inoculated]. CI can range from + 1.0 (complete attraction) to − 1.0 (complete aversion). At least five biological and three technical replicates were kept for each treatment.

### Statistics

The experimental data were checked for normality using the Shapiro-Wilk test. Treatments were either compared pairwise using the *t*-test (as stated in the figure legends) or subjected to one-way ANOVA followed by Tukey’s honest significant difference (HSD)-based multiple comparisons (between different treatments) test in SAS v. 14.1 software.

### Electronic supplementary material

Below is the link to the electronic supplementary material.

**Supplementary Material 1: Table 1.** Oligonucleotides used for RACE-PCR and verifying full-length cDNA sequence; **Table 2.** Oligonucleotides used for RNAi and qPCR analysis; **Table 3.** Putative Mg-ODR-7, Mg-TAX-4, Mg-TAX-4.1, Mg-OSM-9 and Mg-OCR-2 homologues in different nematode species encompassing clades 2, 8, 9, 10, 11 and 12 demonstrating the inter-clade conservation of *M. graminicola* olfactory genes in phylum Nematoda

**Supplementary Material 2: Figure 1.** Schematic alignment of chemosensory gene sequences of *M. graminicola* with that of *C. elegans*; **Figure 2.** Evolutionary relationship of Mg-ODR-7 protein from *M. graminicola* with their corresponding homologues from other nematode species; **Figure 3.** Evolutionary relationship of Mg-TAX-4 protein from *M. graminicola* with their corresponding homologues from other nematode species; **Figure 4.** Evolutionary relationship of Mg-TAX-4.1 protein from *M. graminicola* with their corresponding homologues from other nematode species; **Figure 5.** Evolutionary relationship of Mg-OSM-9 protein from *M. graminicola* with their corresponding homologues from other nematode species; **Figure 6.** Evolutionary relationship of Mg-OCR-2 protein from *M. graminicola* with their corresponding homologues from other nematode species; **Figure 7.** Multiple sequence alignment of dsRNAs corresponding to *Mg-odr-7* (364 bp), *Mg-tax-4* (487 bp), *Mg-tax-4.1* (495 bp), *Mg-osm-9* (495 bp) and *Mg-ocr-2* (623 bp) genes of *M. graminicola*; **Figure 8.** Chemotaxis assay plate showing establishment of the concentration gradient. (A) Instead of test chemical acid fuchsin applied at 1.5 cm distance from the nematode inoculation point. (B) Acid fuchsin diffused through the Pluronic gel from higher to lower concentrations within 40 min to establish an equilibrium. This equilibrium remained in a steady state up to 4 h; **Figure 9.** Representative images show attraction of control J2s towards rice root tip at different time points; **Figure 10.** Comparative penetration potential of control and RNAi worms in rice root at 24 h after inoculation; **Figure 11.** Nematode-infected rice plants inside the covered Petri dishes (110×25 mm) containing Pluronic gel medium at 16 days after inoculation; **Figure 12.** Set up of chemotaxis assay is schematically represented

## Data Availability

The data sets supporting this article are included in the article and in the additional files. The NCBI Genbank accession numbers for the cloned genes are OQ445600, OQ445601, OQ445602, OQ445603 and OQ445605.
