# Supplementary material for: Molecular and functional characterization of chemosensory genes from the root-knot nematode Meloidogyne graminicola
Source: BMC Genomics. 2023 Dec 6;24:745. doi: 10.1186/s12864-023-09864-7 (PMC10698902; doi:10.1186/s12864-023-09864-7)
Supplement: Supplementary file 1 — Supplementary Material 1: Table 1. Oligonucleotides used for RACE-PCR and verifying full-length cDNA sequence; Table 2. Oligonucleotides used for RNAi and qPCR analysis; Table 3. Putative Mg-ODR-7, Mg-TAX-4, Mg-TAX-4.1, Mg-OSM-9 and Mg-OCR-2 homologues in different nematode species encompassing clades 2, 8, 9, 10, 11 and 12 demonstrating the inter-clade conservation of M. graminicola olfactory genes in phylum Nematoda [file 12864_2023_9864_MOESM1_ESM.pdf]

Supplementary Table 1. Oligonucleotides used for RACE-PCR and verifying full-length cDNA sequence. T_m = 60°C

Gene/primer detail	Orientation	Sequence (5'-3')	Purpose
Mg-odr-7 cDNA	Sense	CCTCAAACACAACAGCAGCT	for obtaining first strand cDNA
	Antisense	GGCATTATCATTTATTAGGGTCGAT	
Mg-odr-7 GSP1*	Antisense	AGATGTTGTGCAAATCGGGC	5'-RACE
Mg-odr-7 NGSP1**	Antisense	GCTGAAATTGTTGAACTATTCTGT	
Mg-odr-7 GSP2*	Sense	ACGTGATTATTTCTTTGCGGATG	3'-RACE
Mg-odr-7 NGSP2**	Sense	TTCAGTCAATAACTAGAACAGAAC	
Fragment 1 (start codon – 404 bp)	Sense	ATGGATACAACAGCGCAAACTC	Primer walking for sequencing
	Antisense	AGATGTTGTGCAAATCGGGC	
Fragment 2 (385 bp – 1004 bp)	Sense	GCCCGATTTGCACAACATCT	
	Antisense	TCTTGAGAGGATTCTAAAGGAA	
Fragment 3 (983 bp – stop codon)	Sense	TTCCTTTAGAATCCTCTCAAGA	
	Antisense	CTATTGATTCCATCCAGTAATTC	
Mg-odr-7 cDNA	Sense	ATGGATACAACAGCGCAAACTC	Full-length sequence verification
	Antisense	CTATTGATTCCATCCAGTAATTC	
Mg-tax-4 cDNA	Sense	ACTACTTTAAATGCTCGGCCA	for obtaining first strand cDNA
	Antisense	ACTCTTCTCCTCCTCCTTCTT	
Mg-tax-4 GSP1*	Antisense	CCAATAATAGTAGTAATCTCGATAA	5'-RACE
Mg-tax-4 NGSP1**	Antisense	TCCAGCATAACCATCCTCTTGT	
Mg-tax-4 GSP2*	Sense	CAAATATCAAAACGTAAGCCTTCAG	3'-RACE
Mg-tax-4 NGSP2**	Sense	GGATGTTCCCCTTCTGAAGATG	
Fragment 1 (start codon – 612 bp)	Sense	ATGAGGAAATTGCCTCCTTTACG	Primer walking for sequencing
	Antisense	GACATCACGAACTAGCAATCCT	

Fragment 2 (591 bp – 1147 bp)	Sense	AGGATTGCTAGTTCGTGATGTC	
	Antisense	CGTCTGAGCTTGCTATCATG	
Fragment 3 (1128 bp – 1957 bp)	Sense	CATGATAGCAAGCTCAGACG	
	Antisense	CATCATCATCACTGCTACTACT	
Fragment 4 (1936 bp – 2804 bp)	Sense	AGTAGTAGCAGTGATGATGATG	
	Antisense	CCAGCCTGTACTTGATGTTTTA	
Fragment 5 (2783 bp – 3585 bp)	Sense	TAAAACATCAAGTACAGGCTGG	
	Antisense	CATTCCAAGGAATAAACCCATAT	
Fragment 6 (3563 bp – stop codon)	Sense	ATATGGGTTTATTCCTTGGAATG	
	Antisense	TTATGTTGTAAAGGAAAGGAAGTAAAT	
Mg-tax-4 cDNA	Sense	ATGAGGAAATTGCCTCCTTTACG	Full-length sequence verification
	Antisense	ATGTTGTAAAGGAAAGGAAGTAAATAA	
Mg-tax-4.1 cDNA	Sense	CAGCCTCACAAAGTCGATCG	for obtaining first strand cDNA
	Antisense	TCCTCAGTCATCTCCTCTGC	
Mg-tax-4.1 GSP1*	Antisense	GGTTGTTGTTCTACATTAACTTC	5'-RACE
Mg-tax-4.1 NGSP1**	Antisense	AATGTCTCTGCCACTTTCGC	
Mg-tax-4.1 GSP2*	Sense	ATGTTTGTCGTAAAGGAGATATTGG	3'-RACE
Mg-tax-4.1 NGSP2**	Sense	ACTCTTGTAGAAGGAGCCGT	
Fragment 1 (5´UTR – 626 bp)	Sense	TTGTATGATAATAATGTTCAGAATTC	Primer walking for sequencing
	Antisense	CTCTTCCCCGGTTCAGCC	
Fragment 2 (609 bp – 1365 bp)	Sense	GGCTGAACCGGGGAAGAG	
	Antisense	CAAATAACCTGTGCGAGAACG	
Fragment 3 (1345 bp – 2039 bp)	Sense	CGTTCTCGCACAGGTTATTTG	
	Antisense	GCCCATAAATAATCGAACCATT	

Fragment 4 (2018 bp – 3´UTR)	Sense	AATGGTTCGATTATTTATGGGC	
	Antisense	TCATTCTTCTTCTCCAAAATCTTC	
Mg-tax-4.1 cDNA	Sense	TTGTATGATAATAATGTTCAGAATTC	Full-length sequence verification
	Antisense	TCATTCTTCTTCTCCAAAATCTTC	
Mg-osm-9 cDNA	Sense	TGGGATTAGAACAGGAGGTGAG	for obtaining first strand cDNA
	Antisense	AGGAACCTCAAGCGAAGGAA	
Mg-osm-9 GSP1*	Antisense	TGGCCAAACATATCTTCTCCC	5'-RACE
Mg-osm-9 NGSP1**	Antisense	ACGCCATTGAGCTTCATGTT	
Mg-osm-9 GSP2*	Sense	CGCAAATCAACTTTCAATATTACCG	3'-RACE
Mg-osm-9 NGSP2**	Sense	ACTAGCAGGTCAACATCGCA	
Fragment 1 (start codon – 629 bp)	Sense	ATGAAACTTCGTTCATTTTTCTCATG	Primer walking for sequencing
	Antisense	GTAGCCCTAGCATTTACTGAAG	
Fragment 2 (608 bp – 1259 bp)	Sense	CTTCAGTAAATGCTAGGGCTAC	
	Antisense	CGCAAATAAACAACAAAACAAATAC	
Fragment 3 (1235 bp – 1805 bp)	Sense	GTATTTGTTTTGTTGTTTATTTGCG	
	Antisense	GTGTCTAAAGCTGTAGCATAAT	
Fragment 4 (1784 bp – 2513 bp)	Sense	ATTATGCTACAGCTTTAGACAC	
	Antisense	CGGTAATATTGAAAGTTGATTTGCG	
Fragment 5 (2489 bp – stop codon)	Sense	CGCAAATCAACTTTCAATATTACCG	
	Antisense	TCTAGAATCATTAGAAGAAGTAGCA	
Mg-osm-9 cDNA	Sense	ATGAAACTTCGTTCATTTTTCTCATG	Full-length sequence verification
	Antisense	TCTAGAATCATTAGAAGAAGTAGCA	
Mg-ocr-2 cDNA	Sense	TGTATCAAGCTGGAAAAGGGAA	for obtaining first strand cDNA
	Antisense	GAAGTCGCTGGTCTCATTCC	

Mg-ocr-2 GSP1*	Antisense	TTTACATCAGCCCCGTGTTG	5'-RACE
Mg-ocr-2 NGSP1**	Antisense	CCCCTTGCATTAAACAACAACC	
Mg-ocr-2 GSP2*	Sense	GGAAAAGACAATGGGCCCAA	3'-RACE
Mg-ocr-2 NGSP2**	Sense	TGCGTAAAGAACAACAAGCAA	
Fragment 1 (start codon – 635 bp)	Sense	ATGGCTGCTCATGTCCAGC	Primer walking for sequencing
	Antisense	TTTACATCAGCCCCGTGTTG	
Fragment 2 (616 bp – 1322 bp)	Sense	CAACACGGGGCTGATGTAAA	
	Antisense	CATGTTGTAGCAGAGAAAGGTC	
Fragment 3 (1301 bp – 2077 bp)	Sense	GACCTTTCTCTGCTACAACATG	
	Antisense	GACAAGTACTCATATCTCCATA	
Fragment 4 (2056 bp – stop codon)	Sense	TATGGAGATATGAGTACTTGTC	
	Antisense	TAACCAATTCCACCCAACCTTGA	
Mg-ocr-2 cDNA	Sense	ATGGCTGCTCATGTCCAGC	Full-length sequence verification
	Antisense	TAACCAATTCCACCCAACCTTGA	

*GSP, gene specific primer; **NGSP, nested gene specific primer.

Supplementary Table 2. Oligonucleotides used for RNAi and qPCR analysis. T_m = 60°C

Gene (NCBI Accession Number)	Primer orientation	Sequence (5'-3')	Purpose	qPCR efficiency (%)	Standard curve R^2
Mg-odr-7 (OQ445600)	Sense	CCAAAAGAAACAGCCACAATTCC	dsRNA synthesis	NA	NA
	Antisense	CATCCGCAAAGAAATAATCACGT			
	Sense	CACCCTCTGCTGCACTTATAC	qPCR	102.6	0.954
	Antisense	AGGTTGTCTAACTTGTGGTGTT			
Mg-tax-4 (OQ445601)	Sense	ACTGGTTTGACTATATCTGGCAA	dsRNA synthesis	NA	NA
	Antisense	TCACGTCCTTTATGTACAAGCA			
	Sense	GCTCTCTAACGTCTGTGAAGTC	qPCR	103.7	0.968
	Antisense	ATGCTTCCAACATTTCCTACAATC			
Mg-tax-4.1 (OQ445603)	Sense	CAGCCTCACAAAGTCGATCG	dsRNA synthesis	NA	NA
	Antisense	TGTTTCTCTTCCCCGGTTCA			
	Sense	GTCCCAGGTCCTCAACAAAA	qPCR	101.9	0.922
	Antisense	ACGCATTTCCAACTGTTTCC			
Mg-osm-9 (OQ445605)	Sense	AGAGAAGAACCTGTTTGTCAAGA	dsRNA synthesis	NA	NA
	Antisense	TGCAAGTGTCAAAGGGGTAT			
	Sense	TTAGGAGACCGGCAAATGTC	qPCR	105.5	0.945
	Antisense	GTCCGTTACACCTGGGAGAA			
Mg-ocr-2 (OQ445602)	Sense	GGTTGTTGTTTAATGCAAGGGG	dsRNA synthesis	NA	NA
	Antisense	GCAATTGAAGAGGCTTGTCC			
	Sense	GGGGGACCAATTCATACAGA	qPCR	104.6	0.978
	Antisense	GATTTACATCAGCCCCGTGT			
GFP (HF675000)	Sense	AGCGGCACGACTTCTTCA	dsRNA synthesis	NA	NA
	Antisense	GTGTGGACAGGTAATGGTTGT			
*18S rRNA**	Sense	TACCGTCCTAGTCTCAACCA	qPCR	107.8	0.992
	Antisense	AGAACATCTAAGGGCATCACA			
Actin*	Sense	ATGGCAACTGCCGCTTCTTCT	qPCR	106.6	0.989
	Antisense	AGATTCCGGACAACGGAAGCGT			

NA, not applicable; * these housekeeping genes were reported by Chen et al. 2018.

Supplementary Table 3. Putative Mg-ODR-7, Mg-TAX-4, Mg-TAX-4.1, Mg-OSM-9 and Mg-OCR-2 homologues in different nematode species encompassing clades 2, 8, 9, 10, 11 and 12 (Holterman et al. 2006, *Mol. Biol. Evol.* 23:1792-1800) demonstrating the inter-clade conservation of *M. graminicola* olfactory genes in phylum Nematoda. Solid black boxes indicate the presence of candidate homologue, as identified via BLASTp (in NCBI and WormBase Parasite), in selected nematode species. Search queries employed and predicted proteins identified are detailed in the methodology.

	Nematode species	*M. graminicola* olfactory protein homologs				
		ODR-7	TAX-4	TAX-4.1	OSM-9	OCR-2
Clade 2	*Trichuris suis*					■
	Trichuris trichiura				■	
	Trichinella spiralis				■	■
	Trichinella pseudospiralis				■	■
	Trichinella zimbabwensis				■	■
	Trichinella patagoniensis				■	■
	Trichinella nelsoni				■	
	Trichinella papuae				■	■
	Trichinella britovi				■	
	Trichinella murrelli				■	
	Trichinella nativa				■	
Clade 8	*Loa loa*	■	■	■	■	■
	Brugia malayi	■	■	■	■	■
	Brugia pahangi	■	■	■	■	■
	Brugia timori					■
	Onchocerca flexuosa		■	■	■	■
	Onchocerca ochengi	■	■	■		
	Cercopithifilaria johnstoni	■	■	■	■	■
	Toxocara canis	■		■	■	■
	Dirofilaria immitis		■	■	■	
	Acanthocheilonema viteae	■	■	■	■	■
	Thelazia callipaeda				■	
	Enterobius vermicularis	■	■	■	■	
	Wuchereria bancrofti	■		■	■	■
	Dracunculus medinensis		■	■	■	
	Litomosoides sigmodontis		■	■	■	■
	Anisakis simplex					■
Clade 9	*Pristionchus pacificus*	■	■	■	■	■
	Caenorhabditis elegans	■	■	■	■	■
	Caenorhabditis remanei	■	■	■	■	■
	Caenorhabditis brenneri	■	■	■	■	
	Caenorhabditis briggsae	■	■	■	■	■
	Caenorhabditis angaria	■	■	■	■	■
	Caenorhabditis auriculariae	■	■	■	■	
	Caenorhabditis bovis	■	■	■	■	■
	Caenorhabditis nigoni	■	■	■	■	■
	Mesorhabditis sp.		■	■		

	Ancylostoma ceylanicum	■	■	■		■
	Ancylostoma caninum	■				■
	Ancylostoma duodenale		■	■		■
	Necator americanus	■	■	■	■	
	Haemonchus contortus					■
	Haemonchus placei	■	■	■	■	■
	Dictyocaulus viviparus	■			■	
	Angiostrongylus cantonensis		■	■	■	■
	Angiostrongylus costaricensis	■			■	■
	Nippostrongylus brasiliensis			■	■	
	Parelaphostrongylus tenuis				■	
	Cylicostephanus goldi	■				
	Heligmosomoides polygyrus	■				
	Oesophagostomum dentatum	■				
	Diploscapter pachys	■	■	■	■	■
	Auanema sp.	■	■	■	■	■
Clade 10	*Aphelenchoides besseyi*	■	■	■	■	■
	Aphelenchoides bicaudatus		■	■		■
	Aphelenchoides fujianensis				■	■
	Bursaphelenchus xylophilus	■	■	■	■	■
	Bursaphelenchus okinawaensis	■	■	■	■	■
	Strongyloides ratti	■	■	■	■	■
	Strongyloides stercoralis		■			
	Steinernema carpocapsae	■	■	■		■
Clade 11	*Halicephalobus* sp.	■	■	■	■	■
	Acrobeloides sp.		■	■		
Clade 12	*Aphelenchus avenae*	■	■	■	■	■
	Ditylenchus dipsaci			■	■	
	Ditylenchus destructor	■	■	■	■	■
	Meloidogyne graminicola	■	■	■	■	■
	Meloidogyne incognita	■	■	■	■	■
	Meloidogyne javanica	■	■	■	■	■
	Meloidogyne arenaria	■	■	■	■	■
	Meloidogyne enterolobii	■	■	■	■	■
	Meloidogyne hapla	■	■	■	■	■
	Meloidogyne chitwoodi	■		■		■
	Meloidogyne floridensis	■	■	■	■	■
	Heterodera schachtii	■	■	■	■	■
	Heterodera glycines	■	■	■	■	■
	Globodera rostochiensis	■	■	■	■	■
	Globodera pallida	■	■	■	■	■
