# Supplementary material for: Molecular and functional characterization of chemosensory genes from the root-knot nematode Meloidogyne graminicola
Source: BMC Genomics. 2023 Dec 6;24:745. doi: 10.1186/s12864-023-09864-7 (PMC10698902; doi:10.1186/s12864-023-09864-7)

Supplementary Figure 1. Schematic alignment of chemosensory gene sequences of *M. graminicola* with that of *C. elegans*. NCBI accession numbers are provided in parentheses. Percent identity between different sequences are shown. Numbers indicate amino acid sequence coordinates.

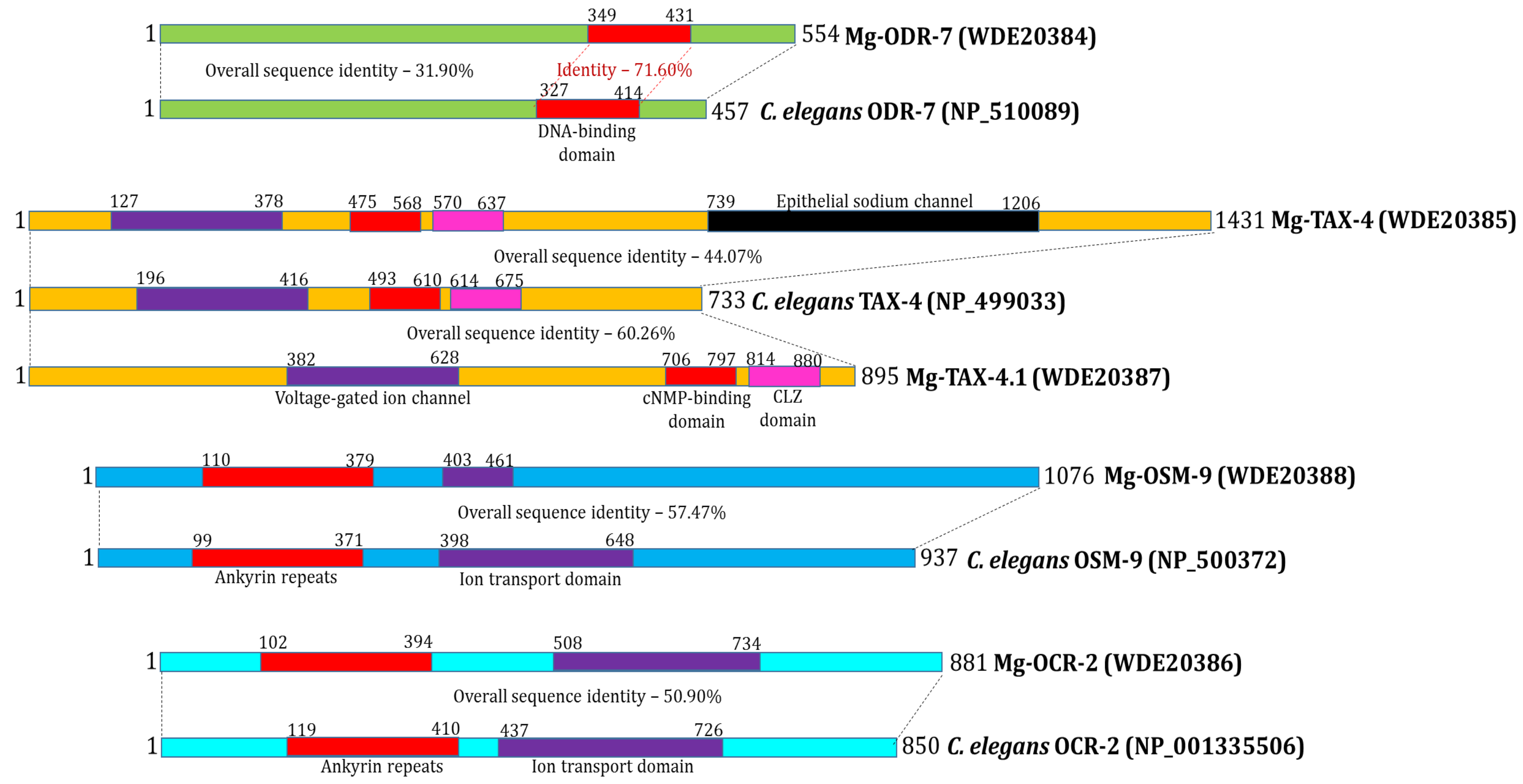

Supplementary Figure 2. Evolutionary relationship of Mg-ODR-7 protein from *M. graminicola* with their corresponding homologues from other nematode species. The phylogenetic tree was constructed in MEGA6 software using Maximum Likelihood method based on Tamura 3-parameter model. Bootstrap consensus was inferred from 1000 replicates and branches corresponding to < 70% replicates were collapsed. NCBI accession numbers and WormBook Parasite gene identifiers of different entries are provided in parentheses. All gaps and missing data positions were eliminated after sequence alignment. *Drosophila melanogaster* sequence for the corresponding protein was used as the out-group (marked with ● and red text). Entries in green, red, blue and purple correspond to the plant-parasitic, animal-parasitic, free-living and fungivorous nematodes, respectively.

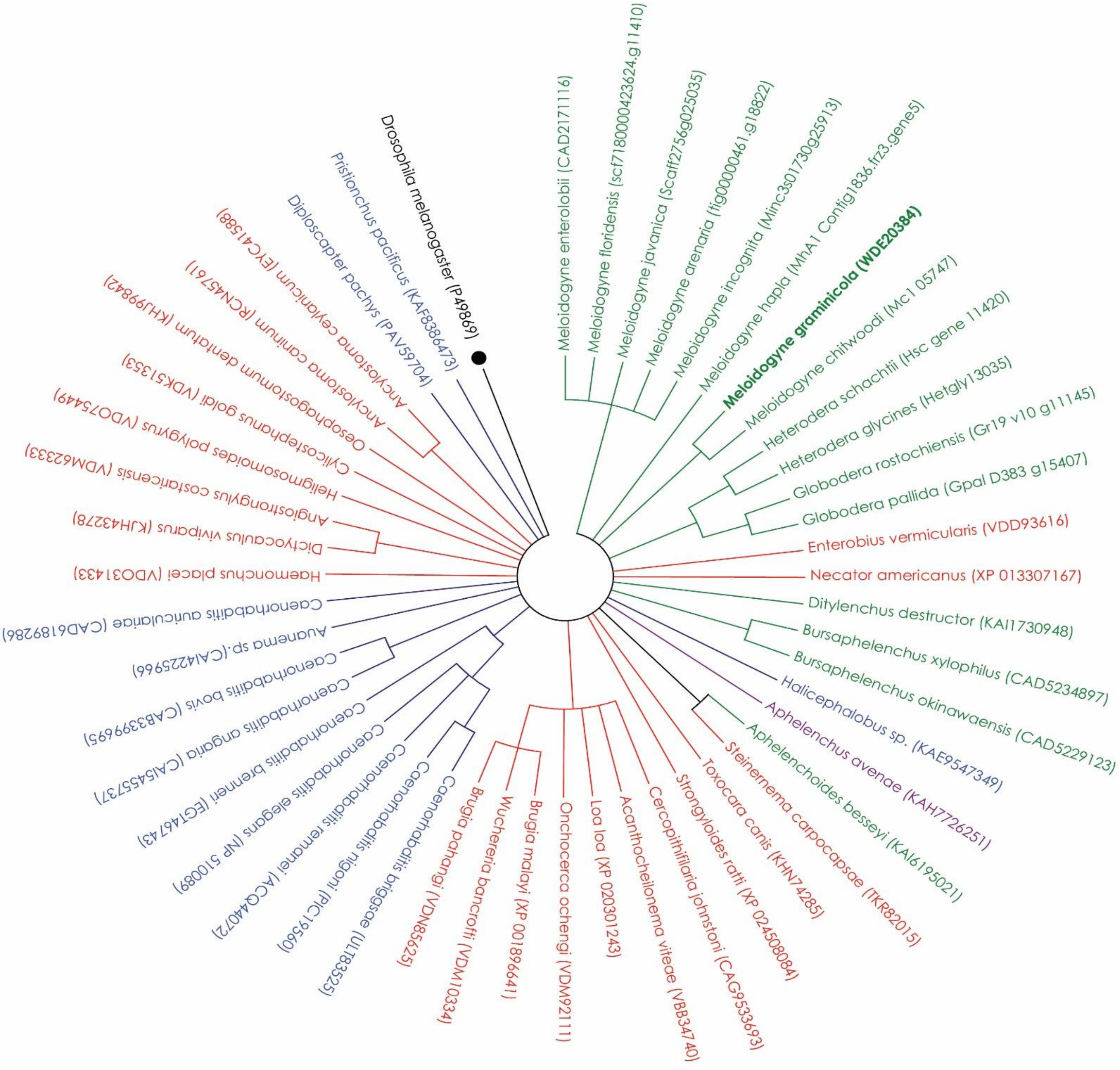

Supplementary Figure 3. Evolutionary relationship of Mg-TAX-4 protein from *M. graminicola* with their corresponding homologues from other nematode species. The phylogenetic tree was constructed in MEGA6 software using Maximum Likelihood method based on Tamura 3-parameter model. Bootstrap consensus was inferred from 1000 replicates and branches corresponding to < 70% replicates were collapsed. NCBI accession numbers and WormBook Parasite gene identifiers of different entries are provided in parentheses. All gaps and missing data positions were eliminated after sequence alignment. *Drosophila melanogaster* sequence for the corresponding protein was used as the out-group (marked with ● and red text). Entries in green, red, blue and purple correspond to the plant-parasitic, animal-parasitic, free-living and fungivorous nematodes, respectively.

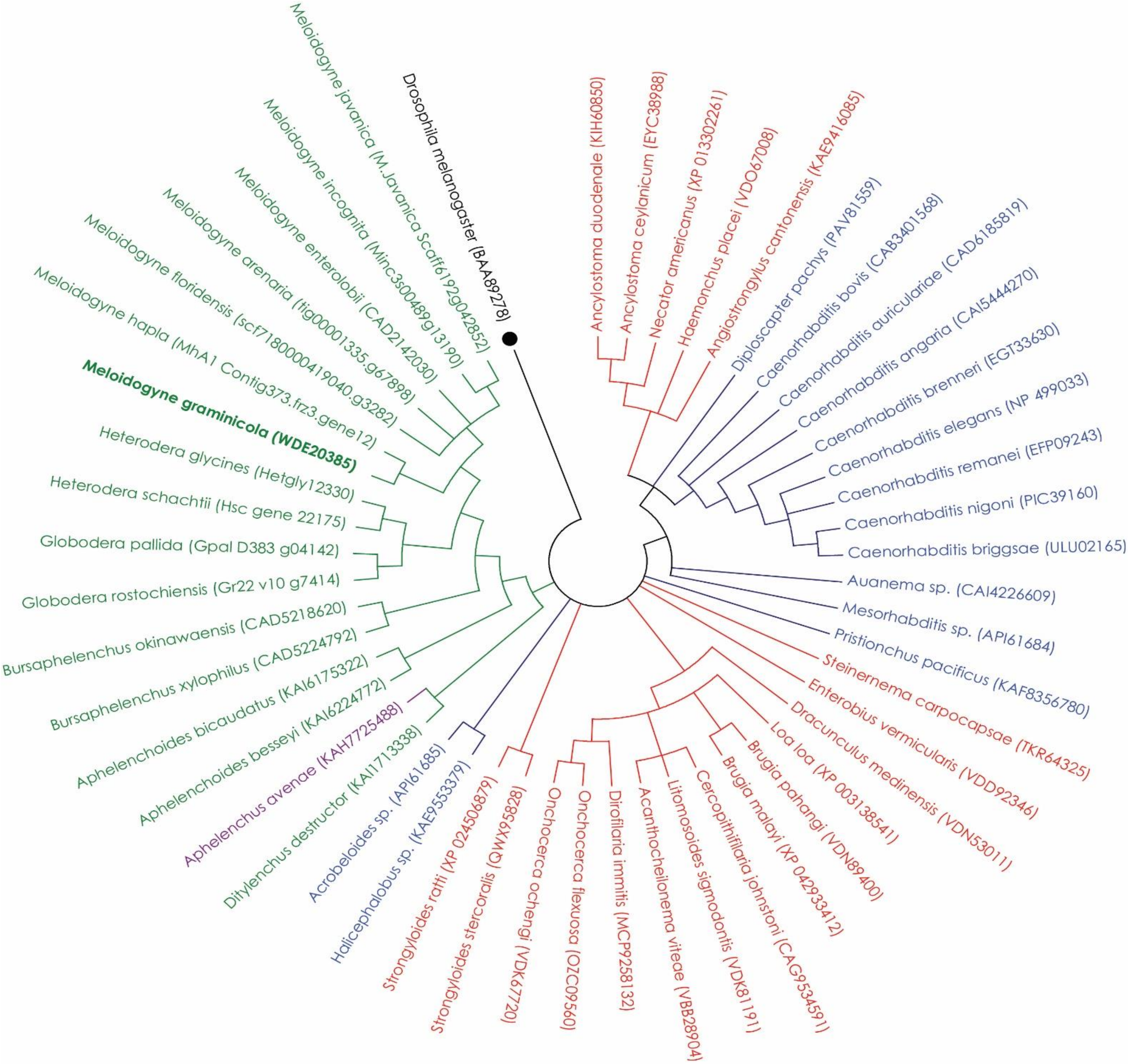

Supplementary Figure 4. Evolutionary relationship of Mg-TAX-4.1 protein from *M. graminicola* with their corresponding homologues from other nematode species. The phylogenetic tree was constructed in MEGA6 software using Maximum Likelihood method based on Tamura 3-parameter model. Bootstrap consensus was inferred from 1000 replicates and branches corresponding to < 70% replicates were collapsed. NCBI accession numbers and WormBook Parasite gene identifiers of different entries are provided in parentheses. All gaps and missing data positions were eliminated after sequence alignment. *Drosophila melanogaster* sequence for the corresponding protein was used as the out-group (marked with ● and red text). Entries in green, red, blue and purple correspond to the plant-parasitic, animal-parasitic, free-living and fungivorous nematodes, respectively.

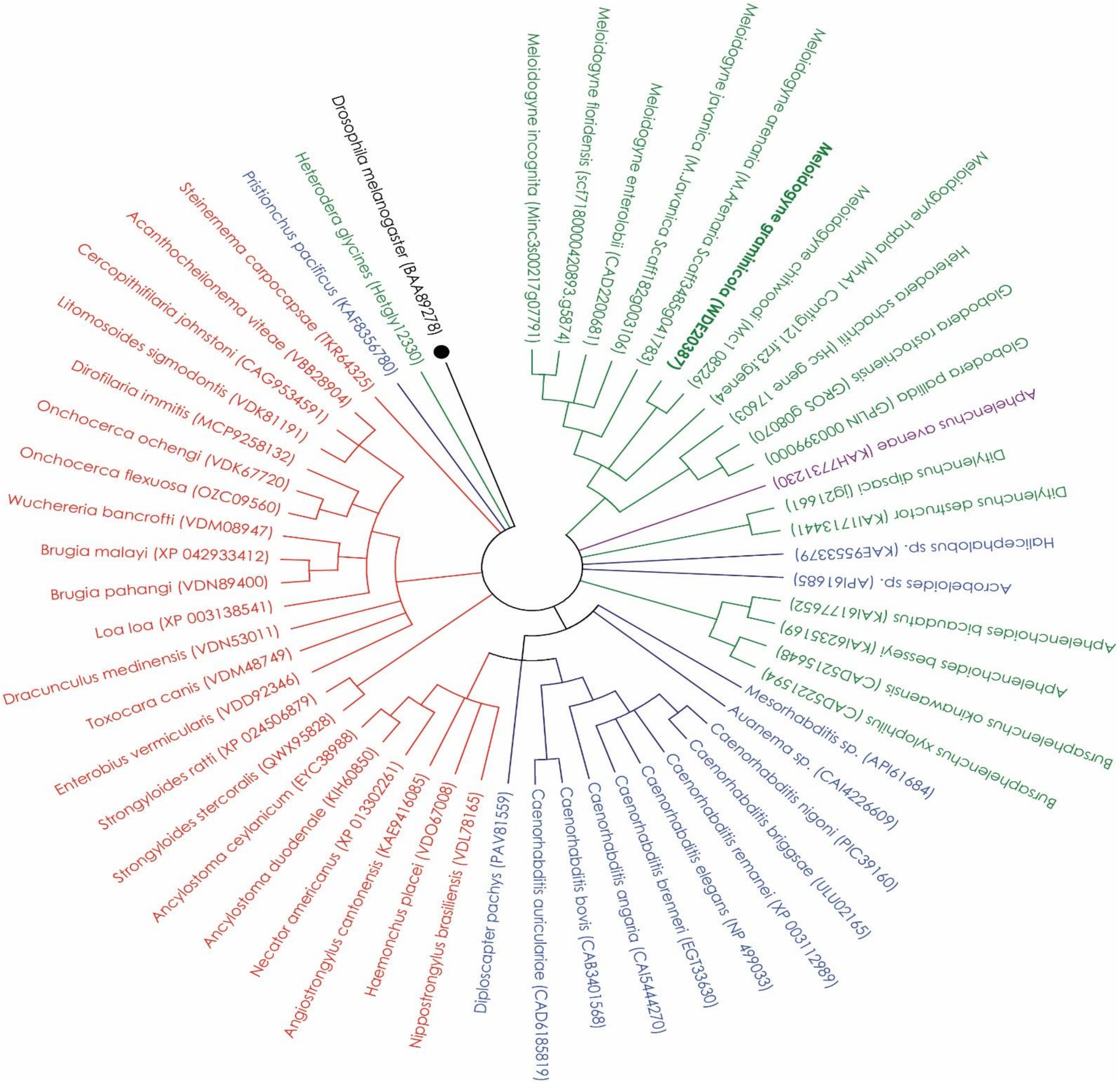

Supplementary Figure 5. Evolutionary relationship of Mg-OSM-9 protein from *M. graminicola* with their corresponding homologues from other nematode species. The phylogenetic tree was constructed in MEGA6 software using Maximum Likelihood method based on Tamura 3-parameter model. Bootstrap consensus was inferred from 1000 replicates and branches corresponding to < 70% replicates were collapsed. NCBI accession numbers and WormBook Parasite gene identifiers of different entries are provided in parentheses. All gaps and missing data positions were eliminated after sequence alignment. *Drosophila melanogaster* sequence for the corresponding protein was used as the out-group (marked with ● and red text). Entries in green, red, blue and purple correspond to the plant-parasitic, animal-parasitic, free-living and fungivorous nematodes, respectively.

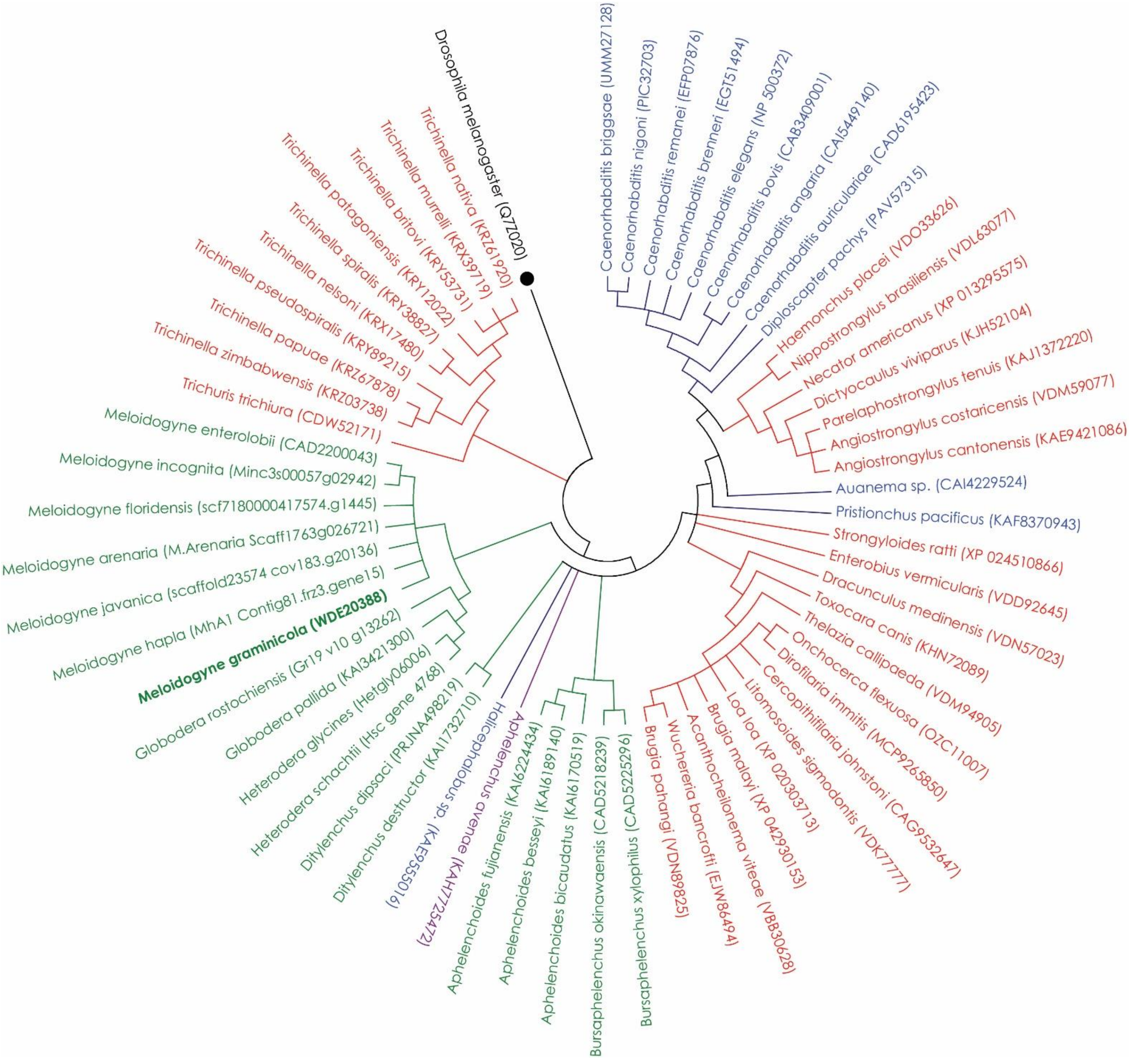

Supplementary Figure 6. Evolutionary relationship of Mg-OCR-2 protein from *M. graminicola* with their corresponding homologues from other nematode species. The phylogenetic tree was constructed in MEGA6 software using Maximum Likelihood method based on Tamura 3-parameter model. Bootstrap consensus was inferred from 1000 replicates and branches corresponding to < 70% replicates were collapsed. NCBI accession numbers and WormBook Parasite gene identifiers of different entries are provided in parentheses. All gaps and missing data positions were eliminated after sequence alignment. *Drosophila melanogaster* sequence for the corresponding protein was used as the out-group (marked with ● and red text). Entries in green, red, blue and purple correspond to the plant-parasitic, animal-parasitic, free-living and fungivorous nematodes, respectively.

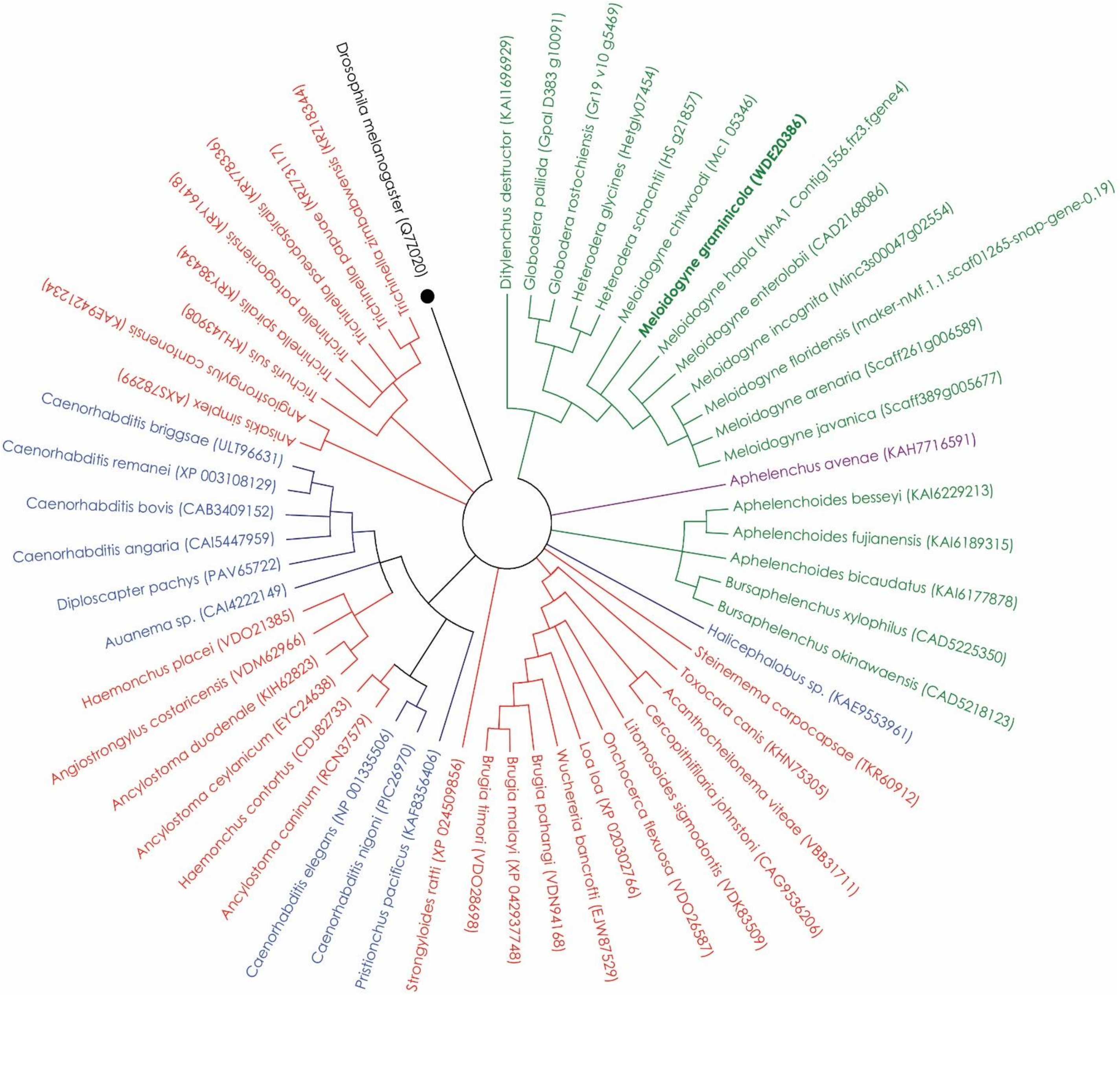

Supplementary Figure 7. Multiple sequence alignment of dsRNAs corresponding to *Mg-odr-7* (364 bp), *Mg-tax-4* (487 bp), *Mg-tax-4.1* (495 bp), *Mg-osm-9* (495 bp) and *Mg-ocr-2* (623 bp) genes of *M. graminicola* using MultAlin (https://www.multalin.toulouse.inra.fr/) Bioinformatics Resource Portal. Red and blue color indicates high and low consensus nucleotides, respectively.

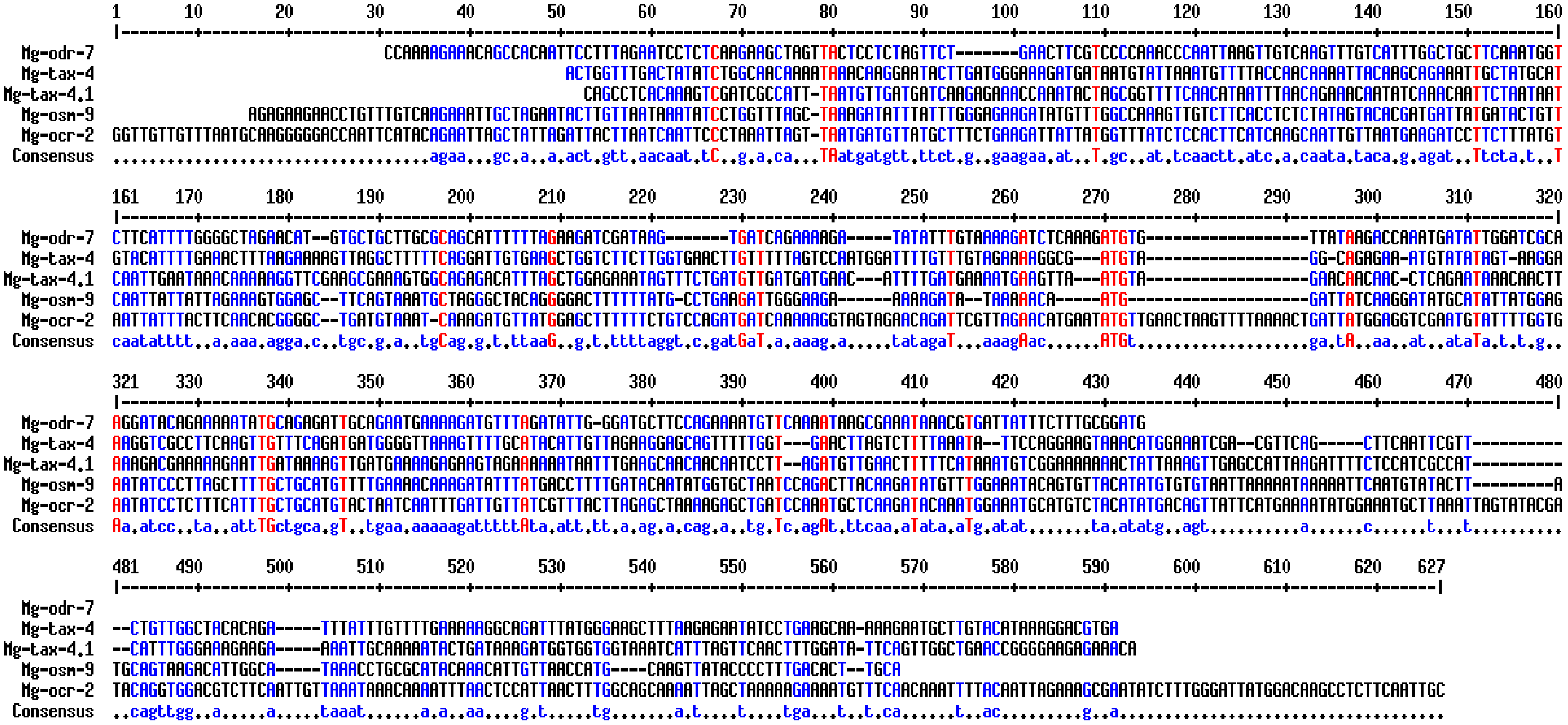

Supplementary Figure 8. (A) Set up of the assay plate (50×10 mm). Instead of test chemical acid fuchsin applied at 1.5 cm distance from the nematode inoculation point. **(B)** Acid fuchsin diffused through the Pluronic gel from higher to lower concentrations within 40 min to establish an equilibrium. This equilibrium remained in a steady state up to 4 h. It is assumed that similar to acid fuchsin, the volatile and nonvolatile compounds may maintain the similar concentration gradient in this assay plate. After 40 min and 4 h, 50 µl each of the test samples was collected from Pluronic gel at 2, 5, 10, and 15 mm distance from the application point of acid fuchsin. Distilled water added to each sample to make up 1 ml which measured for absorbance at 550 nm in a spectrophotometer. Bars indicate mean of 3 replications.

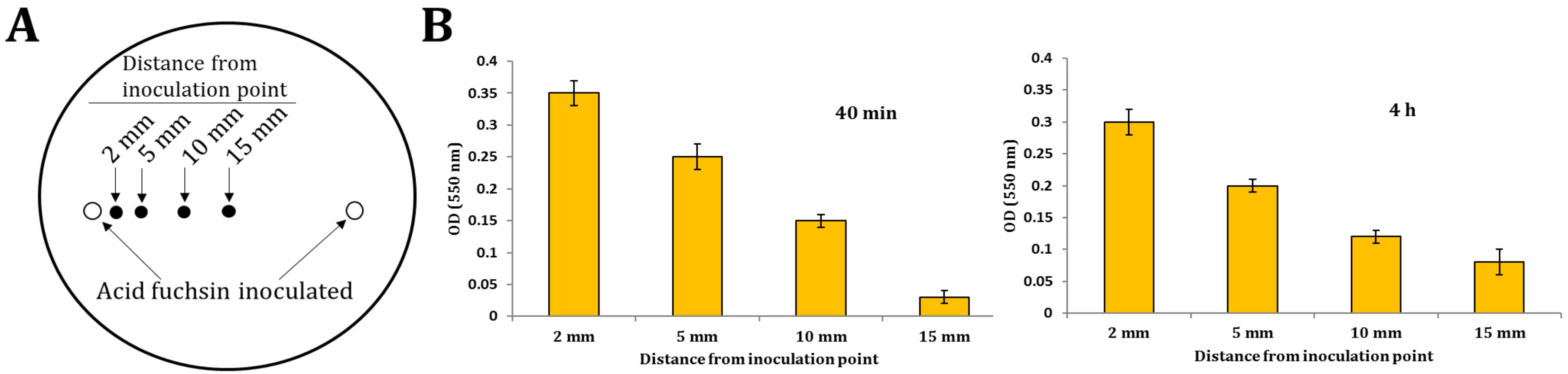

Supplementary Figure 9. Representative images show attraction of control J2s towards rice root tip at different time points.

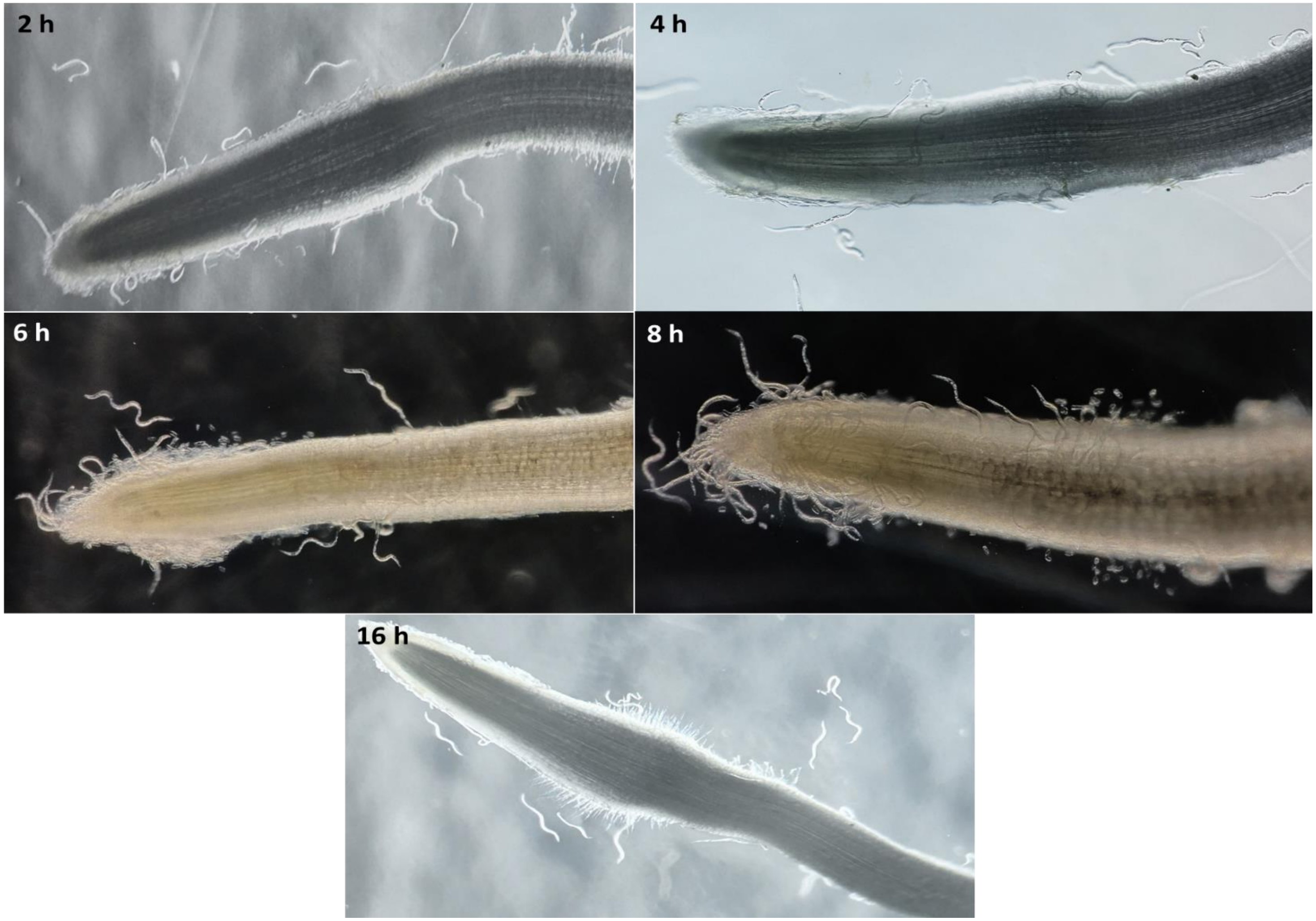

Supplementary Figure 10. Comparative penetration potential of control and RNAi worms in rice root at 24 h after inoculation.

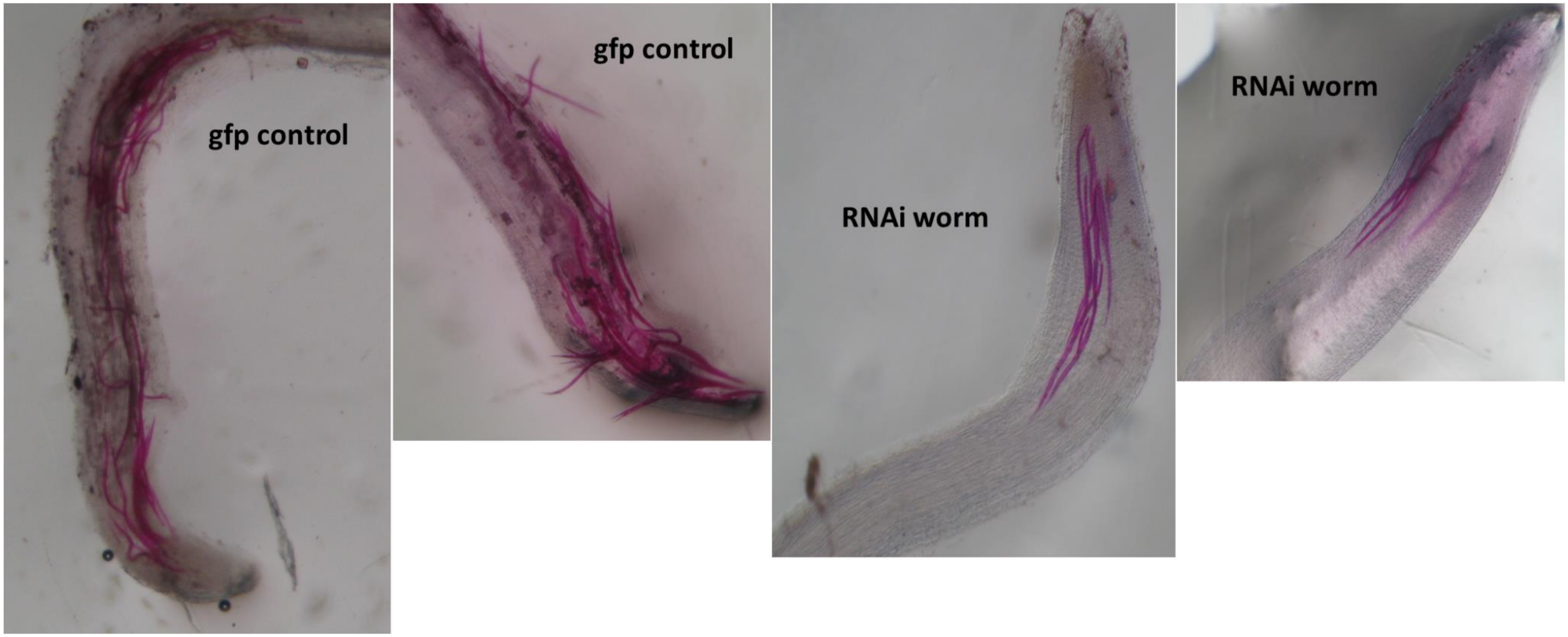

Supplementary Figure 11. Nematode-infected rice plants inside the covered Petri dishes (110×25 mm) containing Pluronic gel medium at 16 days after inoculation.

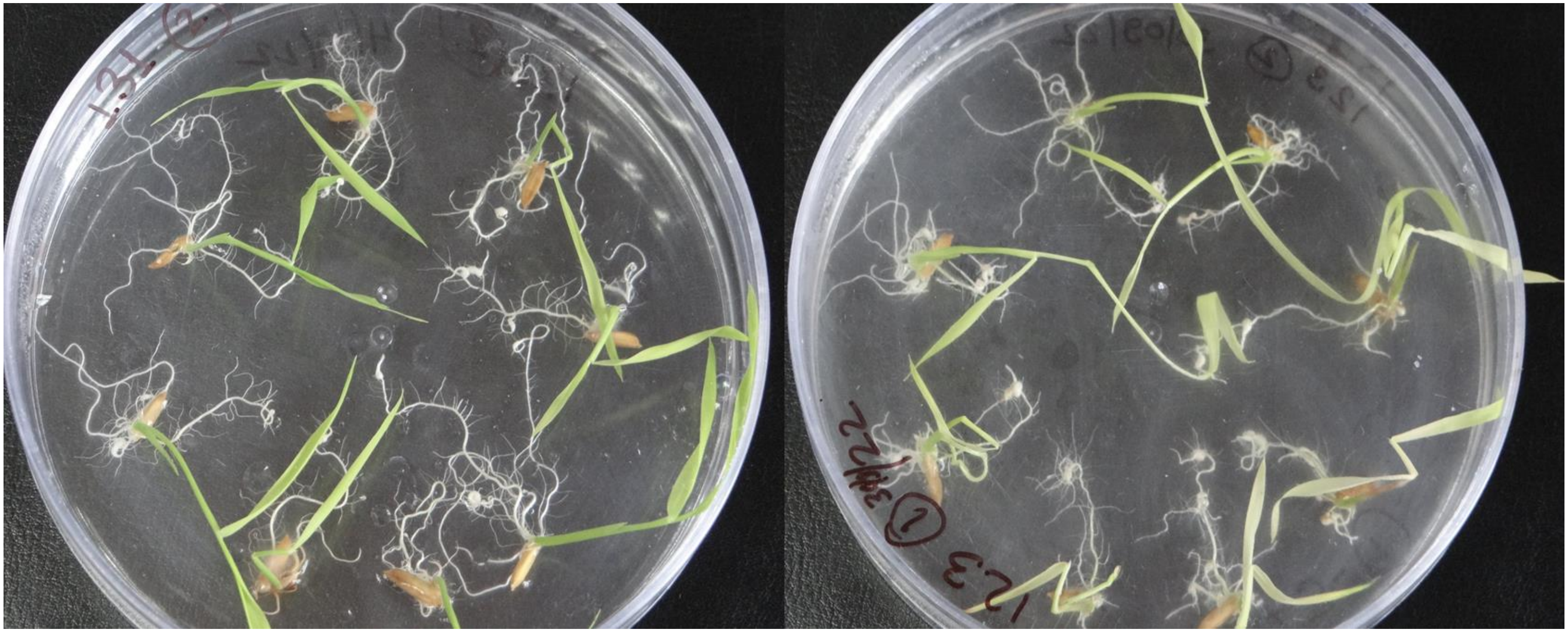

Supplementary Figure 12. Set up of chemotaxis assay is schematically represented. 50×10 mm Petri dish contained 5 ml Pluronic gel media. Nematode inoculation point is 1.5 cm equidistant from test compound or diluent application point. For attraction response, greater number of J2s were accumulated towards test compound side. For repulsion response, greater number of J2s accumulated towards diluent side. Areas demarcated by green and red dashed lines indicate test compound and diluent side, respectively.

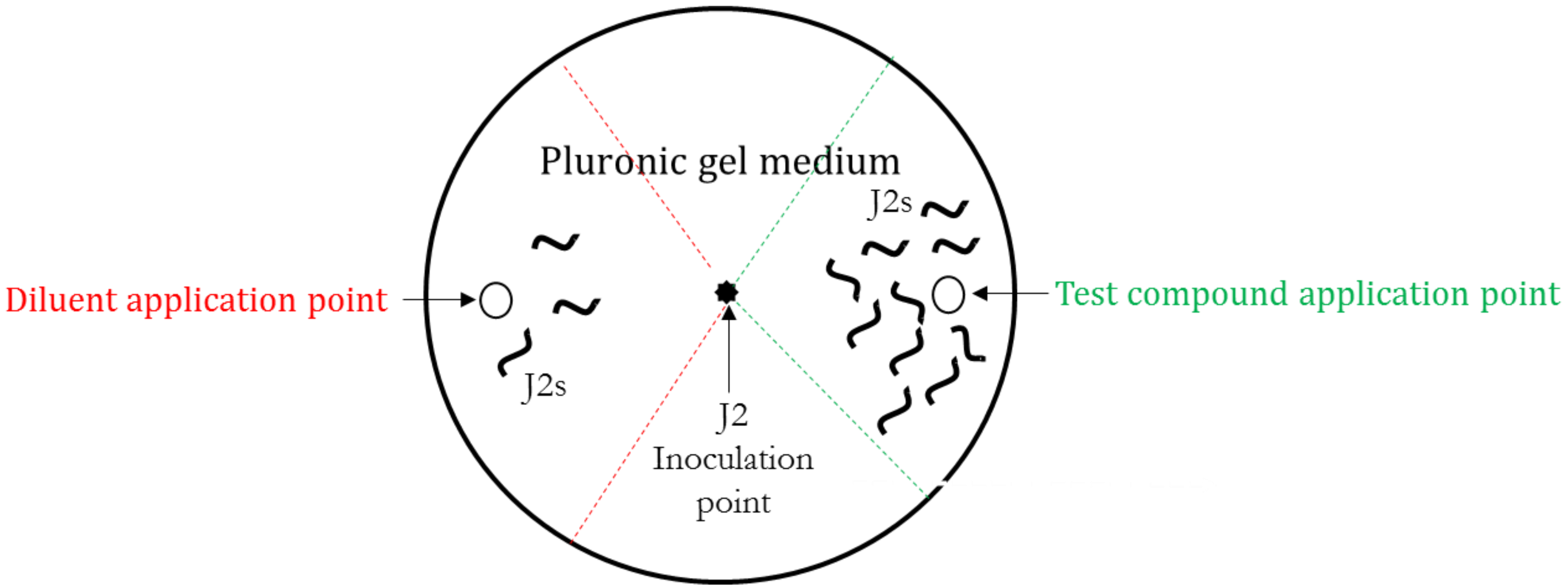

Supplement: Supplementary file 2 — Supplementary Material 2: Figure 1. Schematic alignment of chemosensory gene sequences of M. graminicola with that of C. elegans; Figure 2. Evolutionary relationship of Mg-ODR-7 protein from M. graminicola with their corresponding homologues from other nematode species; Figure 3. Evolutionary relationship of Mg-TAX-4 protein from M. graminicola with their corresponding homologues from other nematode species; Figure 4. Evolutionary relationship of Mg-TAX-4.1 protein from M. graminicola with their corresponding homologues from other nematode species; Figure 5. Evolutionary relationship of Mg-OSM-9 protein from M. graminicola with their corresponding homologues from other nematode species; Figure 6. Evolutionary relationship of Mg-OCR-2 protein from M. graminicola with their corresponding homologues from other nematode species; Figure 7. Multiple sequence alignment of dsRNAs corresponding to Mg-odr-7 (364 bp), Mg-tax-4 (487 bp), Mg-tax-4.1 (495 bp), Mg-osm-9 (495 bp) and Mg-ocr-2 (623 bp) genes of M. graminicola; Figure 8. Chemotaxis assay plate showing establishment of the concentration gradient. (A) Instead of test chemical acid fuchsin applied at 1.5 cm distance from the nematode inoculation point. (B) Acid fuchsin diffused through the Pluronic gel from higher to lower concentrations within 40 min to establish an equilibrium. This equilibrium remained in a steady state up to 4 h; Figure 9. Representative images show attraction of control J2s towards rice root tip at different time points; Figure 10. Comparative penetration potential of control and RNAi worms in rice root at 24 h after inoculation; Figure 11. Nematode-infected rice plants inside the covered Petri dishes (110×25 mm) containing Pluronic gel medium at 16 days after inoculation; Figure 12. Set up of chemotaxis assay is schematically represented [file 12864_2023_9864_MOESM2_ESM.pdf]
